# Innate and learned odor-guided behaviors utilize distinct molecular signaling pathways in a shared dopaminergic circuit

**DOI:** 10.1016/j.celrep.2023.112026

**Published:** 2023-01-25

**Authors:** Nathaniel C. Noyes, Ronald L. Davis

**Affiliations:** 1 Department of Neuroscience, UF Scripps Biomedical Research, 130 Scripps Way #3C2, Jupiter, FL 33458, USA; 2 Lead contact

## Abstract

Odor-based learning and innate odor-driven behavior have been hypothesized to require separate neuronal circuitry. Contrary to this notion, innate behavior and olfactory learning were recently shown to share circuitry that includes the *Drosophila* mushroom body (MB). But how a single circuit drives two discrete behaviors remains unknown. Here, we define an MB circuit responsible for both olfactory learning and innate odor avoidance and the distinct dDA1 dopamine receptor-dependent signaling pathways that mediate these behaviors. Associative learning and learning-induced MB plasticity require rutabaga-encoded adenylyl cyclase activity in the MB. In contrast, innate odor preferences driven by naive MB neurotransmission are rutabaga independent, requiring the adenylyl cyclase ACXD. Both learning and innate odor preferences converge on PKA and the downstream MBON-γ2α′1. Importantly, the utilization of this shared circuitry for innate behavior only becomes apparent with hunger, indicating that hardwired innate behavior becomes more flexible during states of stress.

## In brief

Olfactory learning and innate avoidance are two discrete behaviors that utilize the same *Drosophila* brain region. Noyes and Davis find both behaviors are dopamine dependent but require distinct signaling pathways in the mushroom body. Dopamine utilizes these pathways to bidirectionally modulate the magnitude of neurotransmission to downstream neurons that drive approach.

## Graphical Abstract

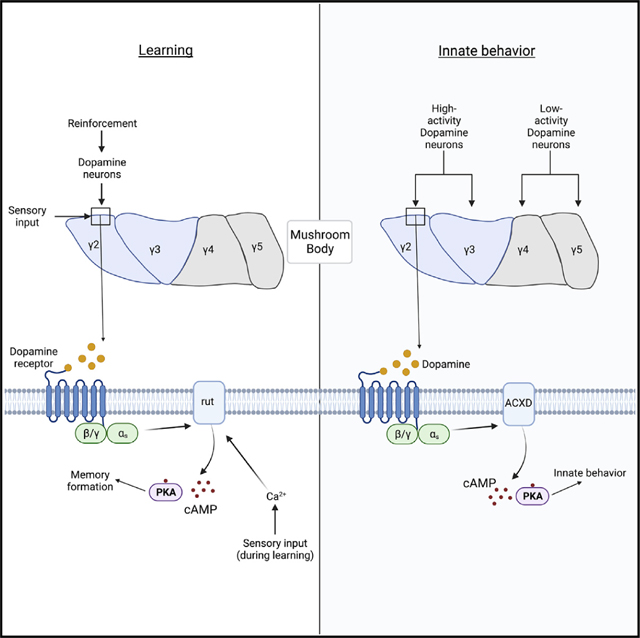

## INTRODUCTION

Shifting internal states and environmental cues require that animals adapt their behavior to survive. Hunger is a powerful internal state that can change innate behavior in response to sensory input.^1–5^ This change in behavior drives attraction to food-related signals and suppresses avoidance to aversive signals. Hunger-dependent suppression of innate avoidance is present across species, causing starved worms to risk desiccation in search of food odors^6^; starved insects to consume food with unpalatable flavors^7^; hungry mice to suppress their aversion to predator odors^8^; and hungry humans to shift risk aversion in the pursuit of food rewards.^9^ This risk taking is hypothesized to be an adaptive response to prioritize behavior that will satisfy the most urgent need.^10^ Specifically, hunger influences olfactory-driven behavior, resulting in modified innate responses to appetitive and aversive odors.^1,11–16^ How hunger affects higher-order brain regions to modify olfactory behavior is not well understood.

In mammals and insects, olfactory information was thought to be processed in parallel such that the computations for hardwired innate preferences and learned associations are performed in separate brain circuits.^17^ However, recent discoveries have revealed that the *Drosophila* mushroom body (MB) is required for innate olfactory behavior as well as its historically defined role in learning.^12,18–20^ The current hypothesis states that both behaviors are driven by cholinergic connections between MB neurons (MBns) and downstream MB-output neurons (MBONs)^21^ and that innervating dopaminergic neurons (DANs) modulate this connection in response to learning and internal state. It is unknown how dopaminergic input to the MB controls these two discrete behaviors.

**Figure 1.**
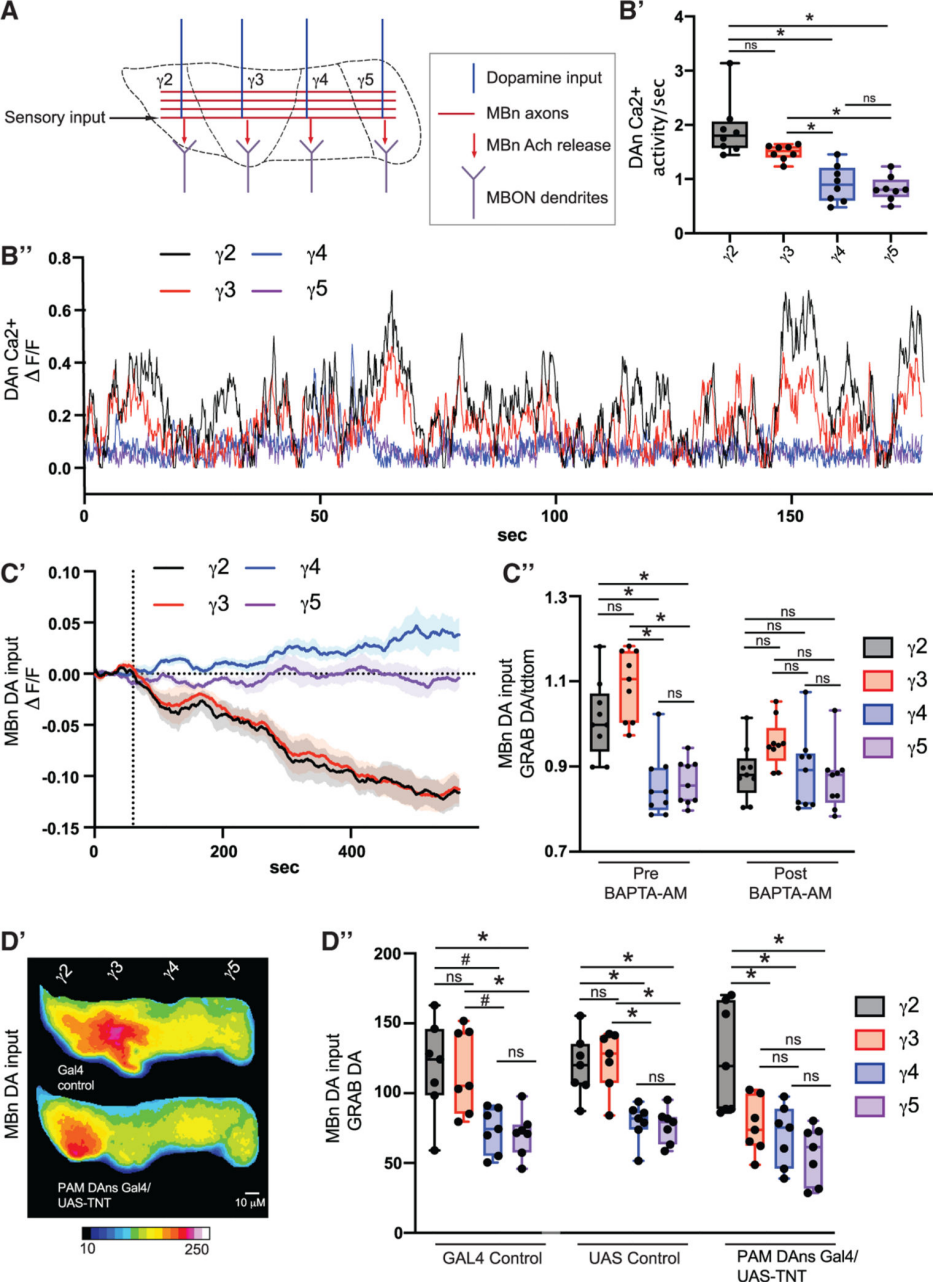
Level of dopamine input to MBn varies across compartments (A) Mushroom body anatomy cartoon. (B) GCaMP-fusionred was expressed in all γ lobe compartment DANs using TH-gal4 and R58E02-gal4. n = 8. (B′) Ca^2+^ activity was calculated by normalizingthe GCaMP signal to the RFP signal. (B″) Example trace of Ca^2+^ activity. (C) The GRAB DA sensor and tdTomato were expressed in MBn using R13F02-gal4. DA activity was calculated by normalizing the GRAB DA signal to the tdTomato signal. n = 9. (C′) GRAB DA/tdTomato values were set to a baseline of zero for each compartment prior to 50 μM BAPTA-AM application at the 1 min mark, indicated by the vertical dotted line. (C″) GRAB DA/tomato activity compared across compartments. (D) The GRAB DA sensor was expressed in MBns using R13F02-lexA, and TNT was expressed in PAM DANs using R58E02-gal4. For all experiments, Gal4 control flies do not contain the UAS element, and UAS control flies do not contain gal4 element. n = 7. (D′) Heatmap of mean time series projection of GRAB DA fluorescence for the whole recording. Scale bar, 10 μm. (D″) GRAB DA signal was compared across compartments. Box-and-whisker plots show the range of individual data points, with the interquartile spread as the box and the median as the line bisecting each box. *p < 0.05. (B-D) One-way ANOVA with Dunn′s test.

MBn axons are compartmentalized with dendrites from non-overlapping MBONs and axons from non-overlapping DANs innervating specific MB compartments (Figure 1A).^22,23^ The MB receives constant and compartmentalized DA input, which is thought to modify MBn-MBON connectivity.^24,25^ However, due to a lack of tools to directly measure neurotransmitter levels, a deep understanding of how DA input affects MB cholinergic neurotransmission has remained elusive. Our understanding of DA-mediated MB physiology is limited for at least 2 reasons. (1) Past work has focused on second messenger systems, namely intracellular Ca^2+^ and cAMP signaling.^25–27^ These important biochemical processes within MBn do not reveal how MBn communicate with behaviorally relevant synaptic partners. (2) Prior studies were concentrated on the effects of DA/odor pairings in an attempt to understand associative learning-induced plasticity.^28–31^ This focus ignored the role of DA in non-associative events, which is likely most of DA actions. The recent development of genetically encoded optical neurotransmitter sensors^32,33^ has allowed us to thoroughly investigate the relationship between DA and acetylcholine (ACh) neurotransmission and the intraneuronal biochemical signaling pathways mediating this relationship.

Here, we demonstrate that DA controls MB ACh neurotransmission and the subsequent hunger-dependent decrease in innate olfactory avoidance. Both learning- and state-dependent odor avoidance require DA signaling through the dDA1 (also known as Dop1R1) dopamine receptor but diverge in downstream molecular signaling pathways. dDA1-dependent learning drives a depression in odor responses that require the adenylyl cyclase rutabaga (rut), while dDA1-dependent odor avoidance requires the facilitation of odor responses through signaling by the adenylyl cyclase ACXD. These divergent signaling pathways reconverge on PKA and the downstream MBON-γ2α′1. We discovered that this MBON is part of a γ2 MB, PPL1-γ2α′1, and MBON-γ2α′1 circuit that is required for state-dependent odor avoidance. Blocking this single MBON eliminates state-dependent odor avoidance and impairs memory,^34^ revealing that both learning and innate odor behaviors converge on MBON-γ2α′1.

## RESULTS

### The level of dopamine input to MBn varies across compartments

To understand the physiological and behavioral effects of DA, we first measured the compartment-specific DA activity and the response of the MB target regions. The MB γ lobe is subdivided into 5 compartments, γ1–5. Each compartment is innervated by axons from non-overlapping DANs.^22,23^ The γ2–5 compartments lay in the same anterior to posterior plane, making it an ideal region to observe compartmentalized physiological differences by *in vivo* functional imaging (Figure 1A). A prior study reported that DANs innervating *γ2* and γ3 display higher activity than γ4 and γ5.^25^ We confirmed this result using GCaMP6m-fusionred expressed in the DANs that innervate the MB γ lobe. This fused sensor allows us to eliminate any spurious signal due to movement or noise by normalizing the GCaMP responses to the fusionred signal. Our results are consistent with the observation that ongoing DAN activity in the *γ2* and γ3 neuropil is elevated relative to that in in γ4 and γ5. (Figures 1B′ and 1B″). We tested whether this patterned pre-synaptic Ca^2+^ activity translates to a difference in DA release, and ultimately DA input, onto MBn. We directly tested this by expressing the GRAB dopamine (DA) sensor^33^ in MBn. The GRAB DA signal was normalized to tdTomato, which was co-expressed with the sensor. We discovered that DA input is significantly elevated in γ2and γ3relative to γ4and γ5(Figure 1C″). Silencing neuronal activity with bath application of the cell-permeable Ca^2+^ chelator BAPTA-AM dramatically reduced the DA signal in γ2 and γ3 and not γ4 and γ5 (Figure 1C′), eliminating the differences across the compartments (Figure 1C″). Genetically blocking neurotransmitter release via expression of tetanus toxin light chain (TNT) specifically in PAM DANs, which include the DANs innervating γ3, γ4, and γ5, significantly reduced DA input to γ3 but not γ4andγ5 Figures 1D′ and 1D″). These data strongly support the conclusion that patterned DAN activity results in patterned input of DA such that the MB compartments γ2 and γ3 receive high DA relative to γ4 and γ5.

### DA controls MBn neurotransmission

**Figure 2.**
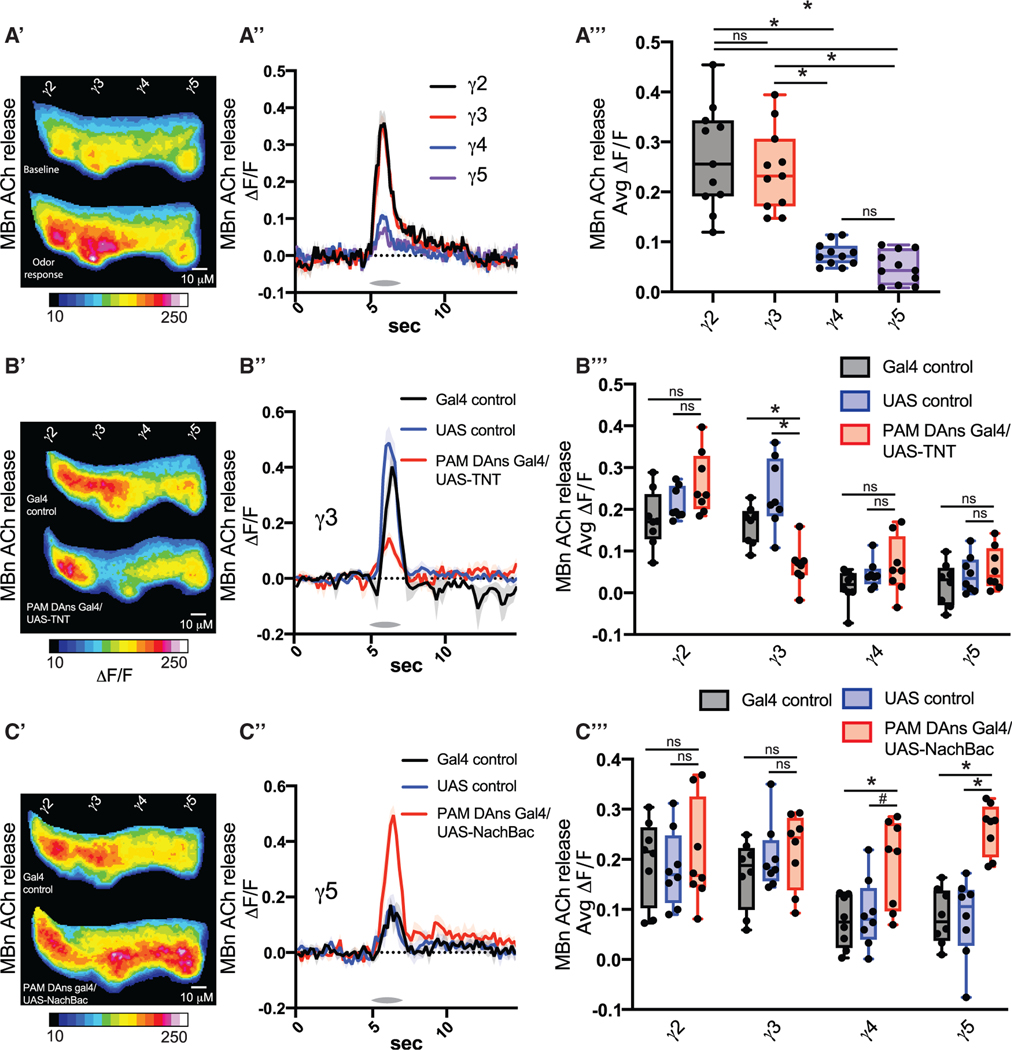
Dopamine controls MBn neurotransmission (A) The GRAB ACh sensor was expressed in MBns using R13F02-gal4. One second of odor (MCH) was delivered at 5 s. n = 11. (A′) Mean time series projection of GRAB ACh fluorescence for baseline, across 2 s prior odor delivery; for odor response, across 2 s starting at odor delivery. Scale bar, 10 μm. (A″) Traces show the average response (±SEM, shading) across all flies tested. (A″′) Average odor responses were quantified for each compartment. (B) The GRAB ACh sensor was expressed in MBns using R13F02-LexA, and TNT was expressed in PAM DANs using R58E02 gal4. One second of odor (MCH) was delivered at 5 s. n = 8. (B′) Mean time series projection of GRAB ACh fluorescence for odor responses, across 2 s starting at odor delivery. Scale bar, 10 μm. (B″) Traces show the average odor (MCH) response (±SEM) across all flies tested in γ3. (B″′) Average odor (MCH) responses were quantified for each compartment. (C) The GRAB ACh sensor was expressed in MBns using R13F02-LexA, and NachBac was expressed in PAM DANs using R58E02 gal4. n = 8. (C′) Mean time series projection of GRAB ACh fluorescence for odor (MCH) responses, across 2 s starting at odor delivery. Scale bar, 10 μm. (C″) Traces show the average response (±SEM) across all flies tested in γ5. (C″′) Average odor responses were quantified for each compartment in γ2–5. Box-and-whisker plots showthe range of individual data points, with the interquartile spread as the box and the median as the line bisecting each box. *p < 0.05. (A) One-way ANOVA with Dunn′s test. (B and C) One-way ANOVA with Kruskal-Wallis test.

Does this pattern of DA input onto the MB affect the properties of the MB in a compartment-specific manner? To address this question, we measured odor-induced cholinergic output from MBn axons in the specific compartments using the GRAB-ACh sensor.^32^ The pattern of ACh release triggered by odor mirrors that of DA input (Figures 2A′–2A″′ and S1A). Across all 4 odors tested, γ2 and γ3 cholinergic release was significantly higher than that from γ4 and γ5. Ca^2+^ activity in DANs innervating γ2 and γ3 are correlated with one another, while the same is true for DANs innervating γ4 and γ5 (Figure S1B′).^25^ Likewise, we found that the magnitude of odor responses in γ2 and γ3 was highly correlated (Figure S1B″). These results indicate that the level of DA input controls the scale of MB odor responses. We tested this possibility by blocking DAN activity in γ3, γ4, and γ5 with TNT (Figures 2B′-B′″ and S1C′-S1C′″). ACh release was significantly reduced in γ3 but not γ4,5 (Figures 2B′″ and S1C′-S1C′″). Conversely, increasing DANs activity in γ3, γ4, and γ5 via expression of the bacterial sodium channel NaChBac increased ACh release in γ4 and γ5 and not in γ3 (Figures 2C′–2C′″ and S2A′-S2A′″). We conclude that compartment-specific DA input levels control the MBn cholinergic output from those compartments.

### The dDA1 DA receptor is required for MBn neurotransmission

**Figure 3.**
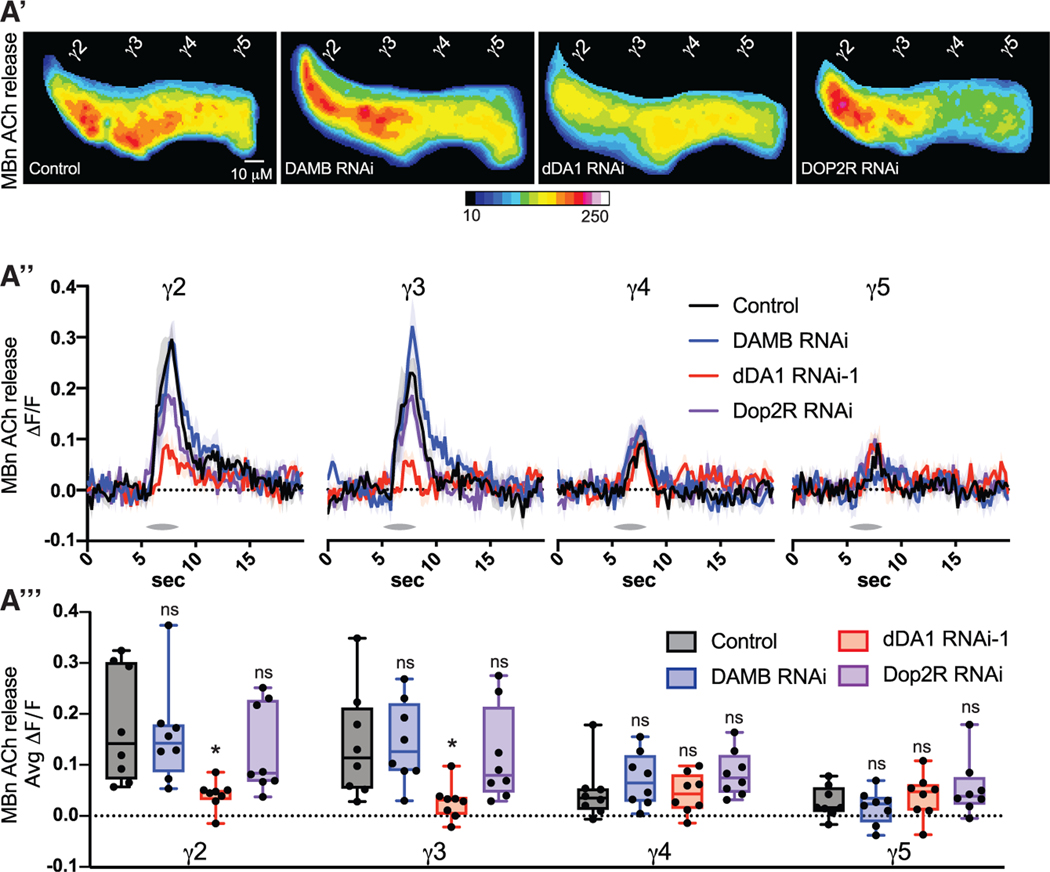
The dDA1 dopamine receptor is required for MBn neurotransmission (A) The GRAB ACh sensor and RNAi transgenes were expressed in MBns using R13F02-gal4. One second of odor (MCH) vapor was delivered at 5 s. n = 8. (A′) Mean time series projection of GRAB Ach fluorescence for odor responses, across 2 s starting at odor delivery. Scale bar, 10 μm. (A″) Traces show the average odor (MCH) response (±SEM) across all flies tested. (A′″) Average odor (MCH) responses were quantified for each compartment in γ2–5. Box-and-whisker plots show the range of individual data points, with the interquartile spread as the box and the median as the line bisecting each box. *p < 0.05. (A) One-way ANOVA with Kruskal-Wallis test.

**Figure 4.**
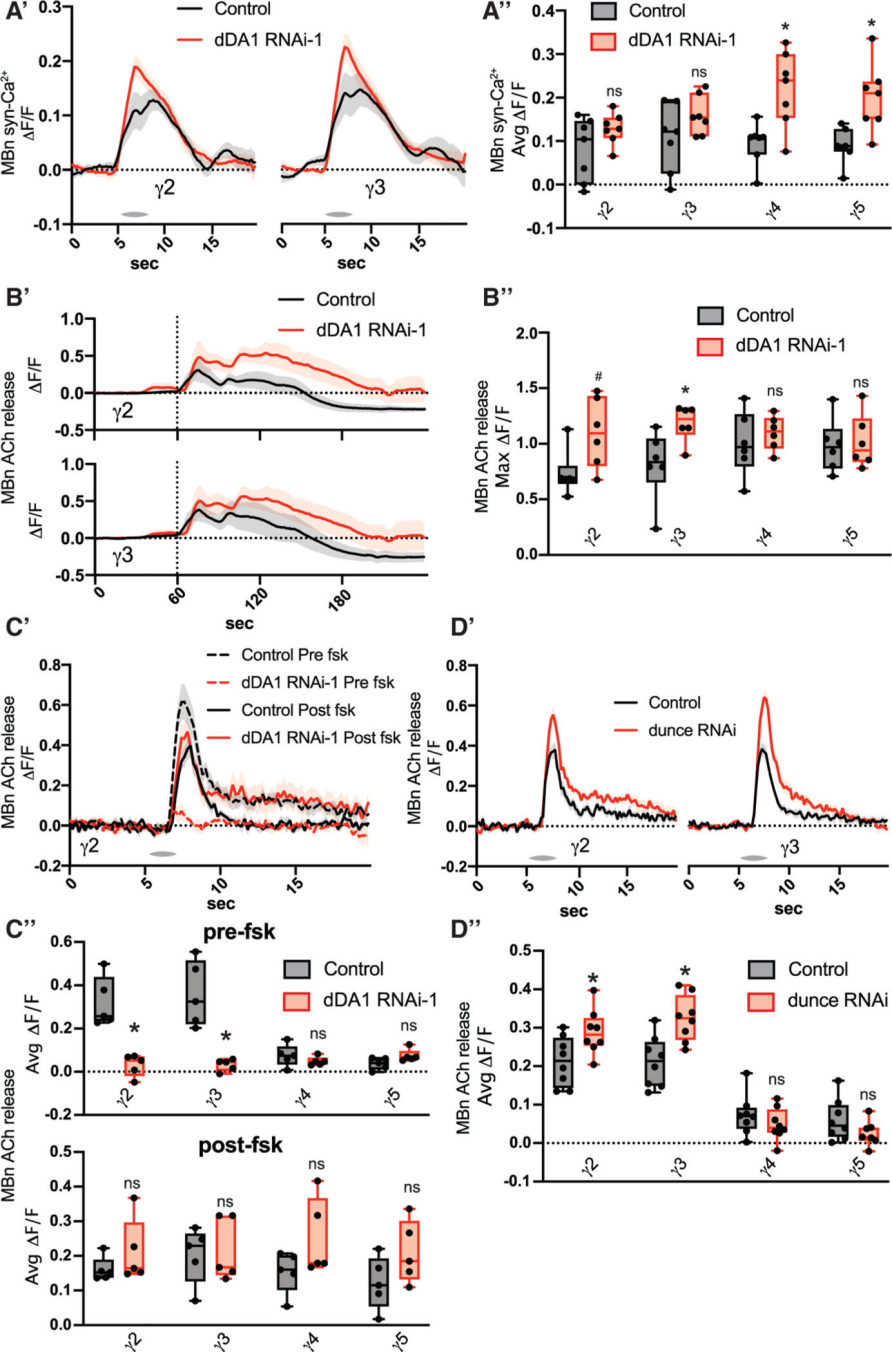
cAMP mediates the effects of dopamine on MBn neurotransmission (A) The syt-GCaMP sensor and RNAi transgenes were expressed in MBns using R13F02-gal4. n = 7. (A^′^) Traces show the average odor (MCH) response (±SEM) across all flies tested in γ2 and γ3. (A″) Average odor (MCH) responses were quantified for each compartment in γ2–5. (B) The GRAB ACh sensor and RNAi were expressed in MBns using R13F02-gal4. Brains were dissected and imaged *ex vivo* in physiological saline. 100 mM KCl was bath applied at the 1 min mark. n = 6. (B′) Traces show the average response (±SEM) across all flies tested in γ2 and γ3. (B″) Maximum responses were quantified for each compartment in γ2–5. (C) The GRAB ACh sensor and RNAi were expressed in MBn using R13F02-gal4. 50 mM forskolin was bath applied between the pre- and post-odor (MCH) response tests. n = 5. (C′) Traces show the average response (±SEM) across all flies tested in γ2. (C′′) Average odor (MCH) responses pre- and post-forskolin treatment were quantified for each compartment in γ2–5. (D) The GRAB ACh sensor and RNAi were expressed in MBns using R13F02-gal4. n = 8. (D′) Traces show the average odor (MCH) response (±SEM) across all flies tested in γ2 and γ3. (D″) Average odor (MCH) responses were quantified for each compartment in γ2–5. Box-and-whisker plots show the range of individual data points, with the interquartile spread as the box and the median as the line bisecting each box. *p < 0.05. (A-D) Mann-Whitney test.

To begin dissecting the molecular pathway by which DA controls MBn cholinergic output, we examined the effects of dopaminergic receptor knockdown. *Drosophila* has two D1-like DA receptors, dDA1 and DAMB, and one D2-like DA receptor, DD2R.^35^ If DA input regulates MBn cholinergic output, we expect that loss of one of these receptors would reduce odor-induced ACh release in γ2 and γ3. Knockdown of MB dDA1 with two different RNAi dramatically reduced odor responses in γ2 and γ3, while γ4 and γ5 were unaffected (Figures 3A′–3A′″, S3A′-S3A′′′, and S4A′-S4A″). Knockdown of DAMB and DD2R did not alter odor responses in any compartment (Figures 3A′–3A′″ and S3A′-S3A′″). This effect of dDA1 knockdown is not due to reduced synaptic Ca^2+^, which would limit ACh release, as the syt-GCaMP signal in response to odor was either unaffected or increased in dDA1 knockdown flies (Figures 4A′, 4A″, and S5A′-S5A′″). In *ex vivo* preparations, we induced depolarization by bath application of 100 mM KCl (Figures 4B′ and 4B″). The GRAB ACh response was not reduced in dDA1 knockdown brains, indicating that dDA1 does not alter ACh available for release and that the GRAB ACh sensor functions normally in dDA1 knockdown flies (Figure 4B″). These results reveal that DA signaling through dDA1 potentiates ACh odor responses independent from cell excitability, synaptic Ca^2+^ levels, and ACh production and trafficking.

### cAMP mediates the effects of DA on MBn neurotransmission

In olfactory learning, MB dDA1 is reported to work through G_a_s, which ultimately drives the production of cAMP.^36–38^ We tested if MB neurotransmission is also cAMP dependent. First, bath application of forskolin (fsk), a drug that increases cAMP levels, rescued the low odor responses due to dDA1 knockdown (Figures 4C′, 4C″, and S5B′-S5B″). Second, knockdown of the dunce-encoded phosphodiesterase, an enzyme that degrades cAMP, increased odor responses in γ2 and γ3 (Figures 4D′, 4D″, and S5C′-S5C″′). These results support a model in which high, compartmentalized DA/dDA1 signaling drives cAMP production, resulting in elevated odor responses in those compartments.

### dDA1 controls state-dependent odor preferences and MBn neurotransmission independent from rut

**Figure 5.**
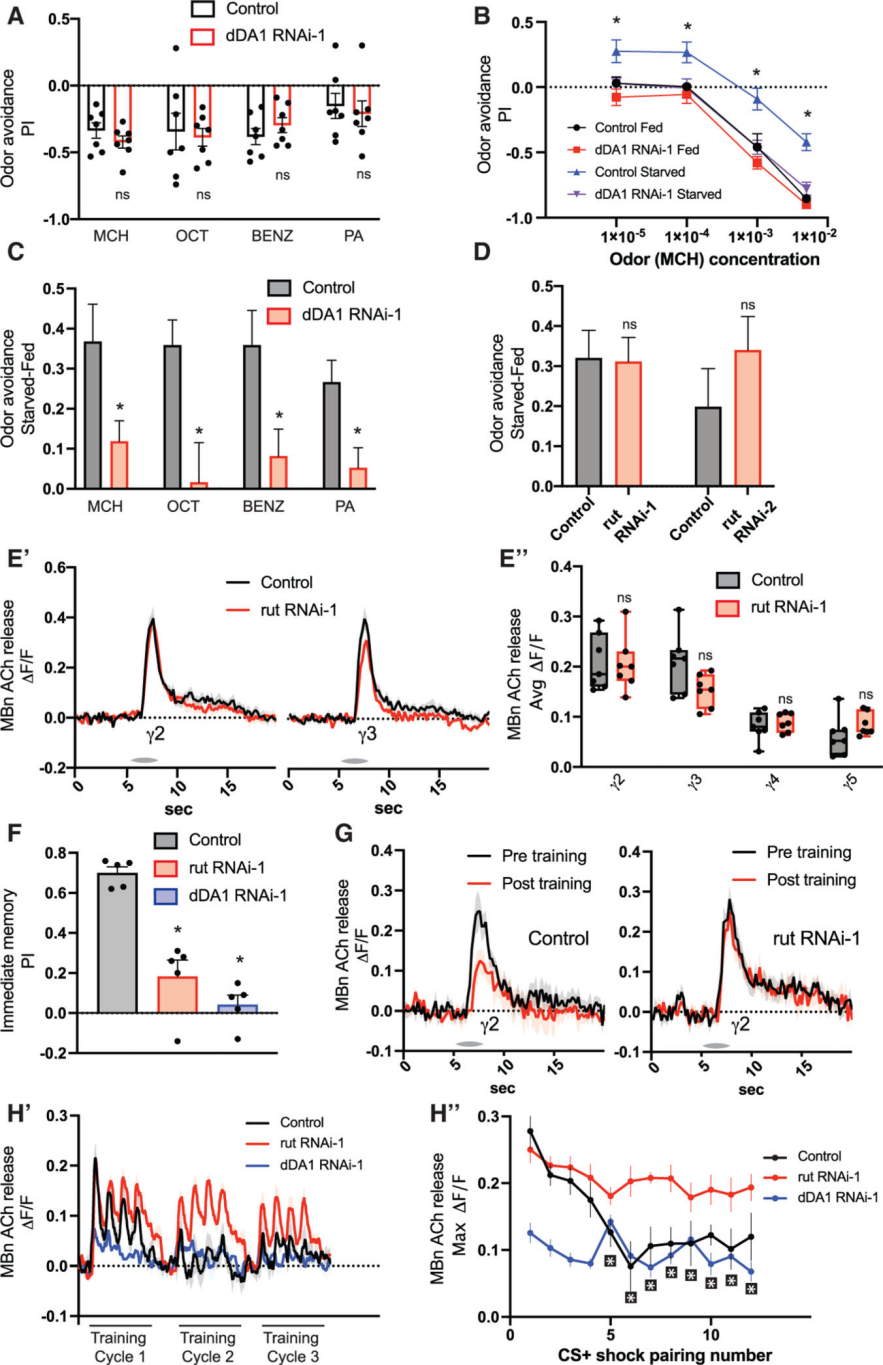
dDA1 controls state-dependent odor preferences and MB neurotransmission independent from rutabaga (A) RNAi was expressed in MBns using R13F02-gal4. The fly′s odor avoidance was tested to MCH, 3-octanol (OCT), benzaldehyde (BENZ), and pentyl acetate (PA). n = 7. (B) Odor avoidance to various doses of MCH. n = 8. (C) Starved-fed odor (MCH) avoidance scores. RNAi was expressed in MBns using R13F02-gal4. n = 8–10. (D) Starved-fed odor (MCH) avoidance scores. RNAi was expressed in MBns using R13F02-gal4. n = 7–8. (E) The GRAB ACh sensor and RNAi were expressed in MBns using R13F02-gal4. n = 7. (E′) Traces show the average odor (MCH) response (±SEM) across all flies tested in γ2 and γ3. (E″) Average odor (MCH) responses were quantified for each compartment in γ2–5. (F) RNAi transgenes were expressed in MBns using R13F02-gal4. Immediate memory was tested following aversive olfactory conditioning. n = 5. (G) The GRAB ACh sensor and rut RNAi were expressed in MBns using R13F02-gal4. Tracesshow the average response (±SEM) in γ2 to the CS+ (OCT) across all flies tested pre- and post-aversive olfactory conditioning. n = 7. (H) The GRAB ACh sensor and RNAi were expressed in MBns using R13F02-gal4. Flies were trained with 3 rounds of 4 CS + odor (OCT) pulses paired with shock followed by 4 CS— odor (MCH) pulses. n = 7. (H′) Traces show the average response (±SEM) to CS+/shock pairings across all flies tested in γ2. The 3 training rounds of CS+/shock were stitched together on the same graph for visualization. (H″) Maximum odor responses were quantified for each CS+/shock pairing in γ2. Box-and-whisker plots show the range of individual data points, with the interquartile spread as the box and the median as the line bisecting each box. *p < 0.05. (A and C-E) Mann-Whitney test. (F) One-way ANOVA with Kruskal-Wallis test.

Because loss of dDA1 has a dramatic effect on MBn odor responses, we reasoned that dDA1 should play a role in olfactory behavior. However, a prior study reported that complete deletion of dDA1 fails to alter odor avoidance.^39^ Indeed, we found that reducing MBn dDA1 had no effect on odor avoidance to four different odors (Figure 5A). Interestingly, starvation produces a shift in odor avoidance to a broad range of odors.^13^ Additionally, MBns and MB-associated neurons are required for attraction to some food odors in starved flies but not fed flies.^19,40–42^ These results led us to test the effect of starvation on odor avoidance of flies with a knockdown of dDA1 in MB. Starvation increased attraction/reduced avoidance to the odor 4-methylcyclohexonal (MCH) over a range of concentrations (Figure 5B). Knockdown of MB dDA1 eliminated this shift in odor avoidance, indicating a prominent role for MB dDA1 in innate, state-dependent odor avoidance. For simplicity, we report the state-dependent shift in odor avoidance by subtracting the odor avoidance performance index (PI) of fed flies from the odor avoidance PI of starved flies (Figure S6A) and the raw values separately (Table S1). We discovered that the change in odor avoidance caused by starvation was eliminated by dDA1 knockdown for each odor tested (Figure 5C). We confirmed this result for the odor MCH with a second dDA1 RNAi as well as with a dDA1 deletion mutant (Figure S6B).

We then probed the molecular pathway downstream of dDA1-dependent MBn cholinergic output and state-dependent odor avoidance by examining the canonical downstream signaling pathway. Associative learning requires MB dDA1 and the downstream adenylyl cyclase encoded by the rut gene.^39,43–46^ Because memory acquisition requires dDA1 and downstream cAMP generation,^27,47^ we tested if rut also functioned downstream of dDA1 to affect state-dependent odor avoidance. Surprisingly, rut knockdown had no effect (Figure 5D). Further, rut knockdown did not reduce ACh neurotransmission in any compartment (Figures 5E′, 5E″, and S6C′-S6C′″). This contrasts with the clear role for dDA1 in state-dependent odor avoidance (Figure 5C) and in MB ACh neurotransmission (Figure 3). These results reveal that MB dDA1 has two important but distinct roles. (1) dDA1 signals through rut to drive olfactory learning. (2) dDA1 mediates state-dependent odor avoidance in a rut-independent but cAMP-dependent (Figures 4C and 4D) manner.

This revelation raises an important question. Since rut is required for learninγ4^3,44,48^ and learning induces MB ACh plasticity,^30,31^ how does rut contribute to learning-induced plasticity of ACh odor responses given that odor-induced ACh responses are independent of rut (Figure 5E)? This question led us to explore the role of dDA1 and rut in learning and learning-induced plasticity. We first confirmed that knockdown of rut and dDA1 in the MB eliminates learning (Figure 5F). We then focused efforts on imaging ACh plasticity in the γ2 compartment in flies with reduced rut and dDA1 expression since aversive training generates a CS+-specific (conditioned stimulus) depression in γ2 ACh output,^30^ and this depression is mirrored in Ca^2+^ responses in the downstream MBON-γ2α′1.^34^ We found that knockdown of rut, while having no effect on odor-induced release of ACh from MBn prior to training, occludes the CS+-specific depression in γ2 ACh output (Figures 5G and S6D′). We observed no change in the response to the CS- or the untrained odor (Figures S6D′′ and S6D′″). These data confirm that rut is required for learning-induced ACh plasticity in γ2. Knockdown of dDA1 resulted in significantly diminished odor responses prior to learning (Figure S6D), prohibiting the assignment of a role for dDA1 in learning-induced synaptic depression. Additional data were obtained by monitoring the ACh odor responses in γ2 across multiple training trials (Figures 5H′, 5H′′, and S6E). Control flies show a rapid depression in CS+ responses as anticipated. By the fifth CS+/shock pairing, the odor responses were significantly lower than the odor response in the first pairing in control flies (Figure 5H′′). In contrast, all subsequent odor responses were not different from the first trial for both rut and dDA1 knockdown flies. Thus, rut is required for associative learning (Figure 5F) and learning-induced acquisition of synaptic plasticity (Figure 5H′′) but does not contribute to dDA1-dependent naive cholinergic odor responses (Figure 5E″) or state-dependent odor avoidance (Figure 5D).

### cAMP signaling is required for state-dependent odor preferences and MBn neurotransmission

**Figure 6.**
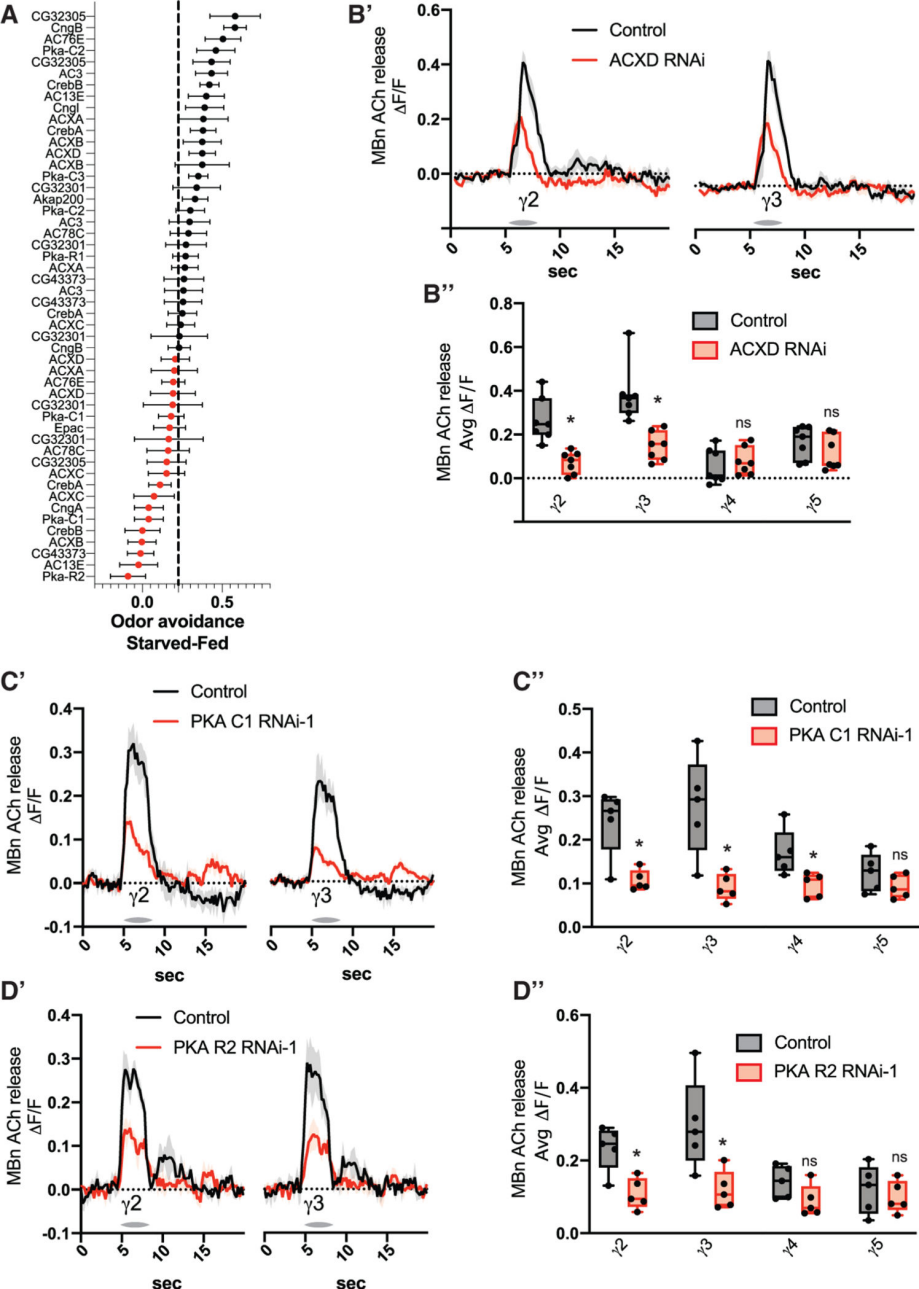
cAMP signaling is required for state-dependent odor preferences and MB neurotransmission (A) Starved-fed odor (MCH) avoidance scores. RNAi was expressed in MBns using R13F02-gal4. n = 6–10. (B) The GRAB ACh sensor and RNAi were expressed in MBns using R13F02-gal4. n = 7. (B′) Traces show the average odor (MCH) response (±SEM) across all flies tested in γ2 and γ3. (B″) Average odor (MCH) responses were quantified for each compartment in γ2–5. (C) The GRAB ACh sensor and RNAi were expressed in MBns using R13F02-gal4. n = 5. (C′) Traces show the average odor (MCH) response (±SEM) across all flies tested in γ2 and γ3. (C″) Average odor (MCH) responses were quantified for each compartment in γ2–5. (D) The GRAB ACh sensor and RNAi were expressed in MBn using R13F02-gal4. (D′) Traces show the average odor (MCH) response (±SEM) across all flies tested in γ2 and γ3. n = 5. (D″) Average odor (MCH) responses were quantified for each compartment in γ2–5. Box-and-whisker plots show the range of individual data points, with the interquartile spread as the box and the median as the line bisecting each box. *p < 0.05. (B-D) Mann-Whitney test.

A rut-independent cAMP signaling pathway responsible for MB ACh output and odor avoidance in naive flies must exist downstream of dDA1 based on the following observations. (1) Increasing cAMP enhances MB ACh release (Figures 4C′–4D′′) and rescues the MB ACh release deficit caused by dDA1 knockdown (Figure 4C″). (2) dDA1-dependent MB ACh release and odor avoidance are independent of rut, the canonical adenylyl cyclase coupled to dDA1 (Figures 5D and 5E″). To identify genes in this proposed pathway, we performed an RNAi behavioral screen measuring the starvation-dependent shift in odor avoidance (Figure 6A). The genes targeted in this screen include adenylyl cyclases and cAMP-sensitive enzymes, ion channels, and transcription factors. The initial screen generated several candidate genes defined by RNAi lines whose starved-fed avoidance difference was lower than the control average (Figure 6A). These RNAi lines were re-screened and statistically compared with their genotypic controls (Figure S7A). The three adenylyl cyclase RNAi lines that passed the re-screen were tested for their effects on MB ACh odor responses (Figures S7B′ and S7B′′). Knockdown of ACXD significantly reduced MB output in γ2 and γ3 in two separate experiments (Figures 6B′, 6B″, and S7B′-S7C). ACXD knockdown produced a reduction of the starvation-dependent odor avoidance shift for three additional odors like that observed with dDA1 knockdown (Figure S7D). The starvation-dependent odor avoidance shift phenotype caused by ACXD knockdown was rescued by ACXD overexpression (Figure S7E). Two additional ACXD RNAi were negative in the original screen, suggesting that these RNAis are less effective at reducing ACXD levels (Figure 6A).

The three additional RNAi lines that passed the re-screen targeted PKA genes: two targeting the catalytic subunit PKA-C1, and one targeting the regulatory subunit PKA-R2 (Figures 6A, S7A). All three reduced MB output in γ2 and γ3 (Figures 6C′–6D′, S7F, S8A, and S8B). Additionally, knockdown of PKA-C1 or PKA-R2 reduced the starvation-dependent odor avoidance shift for three additional odors (Figures S8C and S8D). To confirm the effect of PKA-R2, we tested a second PKA-R2 RNAi line that was not included in the original screen. It also reduced the starvation-dependent odor shift (Figure S8E). We determined that the effects of dDA1, ACXD, and PKA knockdown are not due to developmental effects by using temperature-sensitive temporal control of RNAi expression (Figures S8F and S8G). Finally, we found that adult expression of dDA1, rut, or PKA RNAi impaired learning, while ACXD RNAi had no effect (Figure S8H). Expression of these RNAis during development only had no effect on learning (Figure S8I). Together, these results provide evidence for a dDA1→ACXD→PKA pathway that mediates state-dependent odor avoidance and a dDA1→rut→PKA pathway that mediates olfactory learning.

### Hunger engages the MB circuit in odor avoidance by facilitating γ2α′1 MBON odor responses

We set out to identify a circuit-level mechanism for the starvation-dependent shift in odor avoidance. First, we determined that starvation-dependent odor avoidance requires dDA1 signaling in the MB γ lobe specifically (Figure S9A). Next, we reasoned that if γ MBns are required for starvation-dependent changes in odor avoidance, then odor responses in γ MBns might be altered by starvation. However, starvation had no effect on ACh release from any of the γ MB compartments including γ1–5 or α′1, which contributes ACh to neurons downstream of γ MBn (Figures S9B′ and S9B^″^). Thus, γ MBn ACh serves a permissive role and not an instructive role in starvation-dependent odor avoidance. In other words, γ MBn ACh odor responses are unchanged by starvation but are required for responses downstream of γ MBns that are changed by starvation.

**Figure 7.**
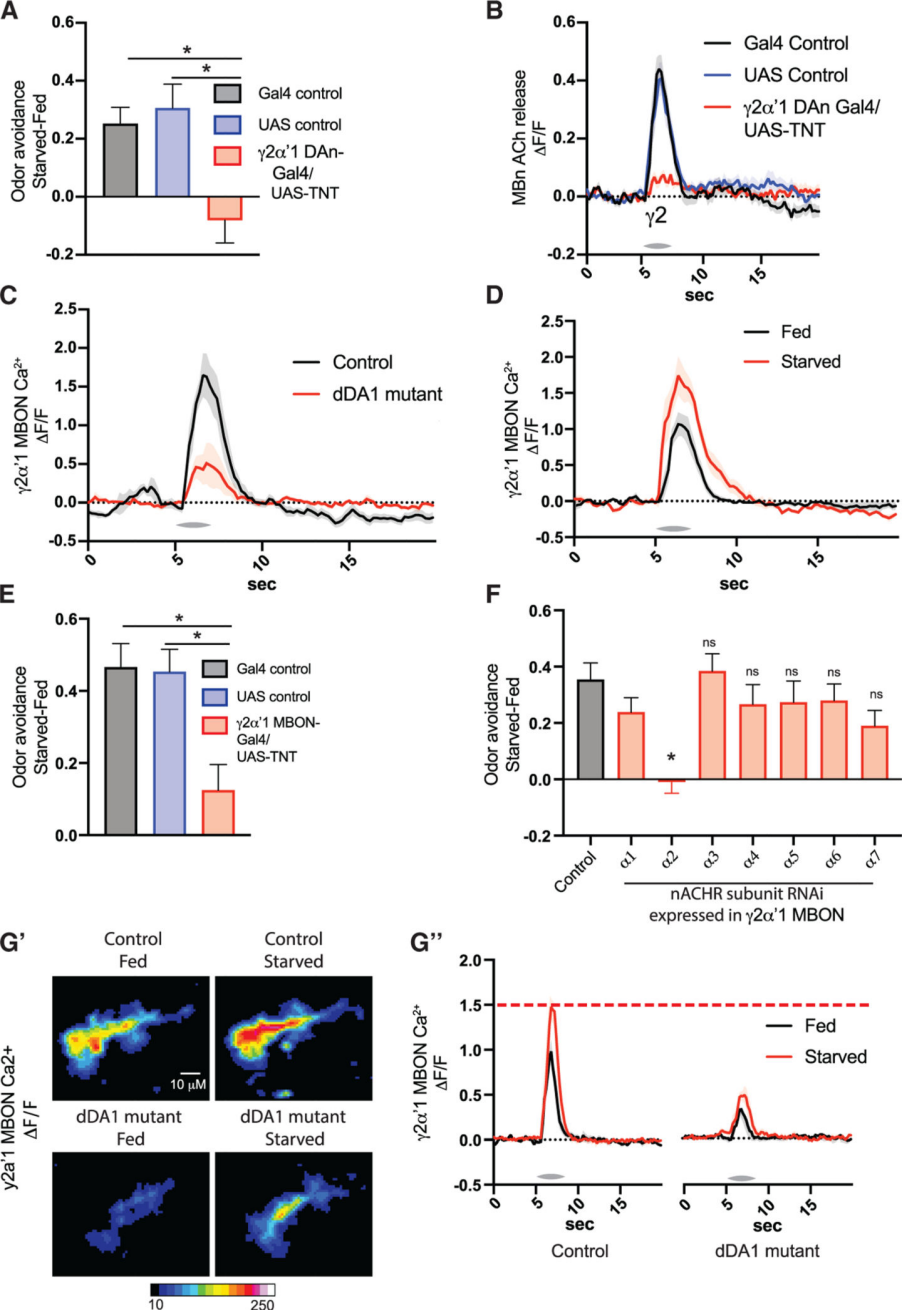
Hunger engages the MB circuit in odor avoidance by facilitating MBON-γ2α′1 odor responses (A) Starved-fed odor (MCH) avoidance scores. TNT was expressed in RRL1-γ2α′1 using MB′99C-split gal4. n = 10. (B) The GRAB ACh sensor was expressed in MBns using R13F02-gal4. TNT was expressed in RRL1-γ2α′1 using MB′99C-split gal4. Traces show the average odor (MCH) response (±SEM) in y2 across all flies tested. n = 8. (C and D) Traces show the average odor (MCH) response (±SEM) in γ2α′1MBON. GCaMR was expressed in MBON-γ2α′1 using R25D′1-gal4 (E) in dDA1 mutant or wild-type (WT) flies, n = 5, and (D) in fed or starved flies, n = 5–6. (E and F) Starved-fed odor (MCH) avoidance scores. (E) TNT was expressed in MBON-γ2α′1 using MB′77C split-gal4. n = 10. (F) RNAi was expressed in γ2α′1 MBONs using MB′77C split-gal4. n = 6. (G) GCaMR was expressed in MBON-γ2α′1 using R25D′1-gal4. Dendrites and axons were imaged and quantified together. n = 7. (G′) Mean time series projection of GCaMR fluorescence for odor (MCH) response, across 2 s starting at odor delivery. Scale bar, 10 μm. (G^″^) Traces showthe average odor (MCH) response (±SEM) across all flies tested.</p/>*p < 0.05. (A and E) One-way ANOVA with Sidak′s test. (F) One-way ANOVA with Dunnett′s test.

To identify the downstream neuron(s) whose responses are altered by starvation and thereby drive starvation-dependent odor avoidance, we mapped the DANs involved in the behavior. We found that THD′ DANs are required, while PAM DANs are not (Figures S9C and S9D). THD′ neurons include DANs that cover some compartments in the MB γ lobe.^22,23^ We predicted that the γ lobe compartments contributing to dDA1-dependent odor avoidance are those innervated by THD′ DANs (i.e., γ1 and γ2). This led to a continued focus on the γ2 compartment. Blocking output from the DAN PPL1-γ2α′1 eliminated the effect of starvation on odor avoidance (Figure 7A), confirming that DA input to the γ2 compartment is critical for this behavior. Odor responses in γ2 MBns require DA input from PPL1-γ2a′1 because blocking this DAN dramatically reduced ACh output from γ2 but not from other compartments (Figures 7B, S10A′, and S10A″). The behaviorally relevant neuron that lies downstream of the γ2 MB compartment is MBON-γ2a′1. We found that loss of dDA1 significantly reduced odor responses in MBON-γ2a′1 (Figures 7C and S10B). Thus, PPL1-γ2a′1 DA signaling through MB γ2 dDA1 is required for MB γ2 ACh/MBON-γ2a′1 odor responses. These data led us to predict that MBON-γ2a′ 1 has odor responses sensitive to starvation and that it is required for starvation-dependent odor avoidance. Confirming these predictions, we found that starvation significantly enhanced odor responses in the MBON-γ2α′1 (Figures 7D and S10C) and that blocking output from this MBON eliminated starvation-dependent odor avoidance (Figure 7E). Further, ACh input to this MBON through the α2 subunit of the nicotinic ACh receptor (nAChR) is required for starvation-dependent odor avoidance (Figure 7F), verifying that ACh input from MBns is required for the effect. MBON-γ2α′1 is appetitive, meaning increased activation results in increased approach/decreased avoidance.^49^ Thus, starvation-induced enhancement of odor responses in MBON-γ2α′1 explains the decrease in odor avoidance observed in starved flies. These results reveal that although starvation does not detectably alter MB ACh transmission, ACh provided by MBns is required for starvation-dependent odor behavior driven by MBON-γ2α′1.

Taken together, these data lead to a model in which MBON-γ2α′1 responses in fed flies are insufficient to contribute to odor avoidance. With starvation, MBON-γ2α′1 responses increase and tilt the balance toward increased odor attraction/decreased odor avoidance. dDA1 in the MB γ2 compartment is required for normal ACh output from γ2. Loss of MB dDA1 dramatically reduces ACh output from γ2 MBns and consequently reduces MBON-γ2α′1 responses in both fed and starved flies. Consistent with this model, we found that although starvation did increase MBON-γ2α′1 responses in flies lacking dDA1, those responses failed to reach the level of odor responses in starved control flies (Figures 7G′, 7G″, and S10D).

## DISCUSSION

Our data reveal the shared use of a discrete circuit for both state-dependent odor-driven behavior and experience-dependent odor learning. The shared components include the upstream DA neurons, the MBn-expressed DA receptor dDA1, and the downstream MBON. Odor response processing for state-dependent behavior and odor learning diverge at the level of the dDA1 receptor-activated adenylyl cyclase, with ACXD employed for innate state-dependent odor driven behavior and rut employed for olfactory learning. The unique activation of rut for olfactory learning is explained by the fact that this adenylyl cyclase functions as a coincidence detector, synergistically responding to both DA receptor activation from the unconditioned stimulus and Ca^2+^ increases due to the conditioned stimulus.^27,47^ ACXD is a transmembrane AC that is expressed in a number of tissues including the brain (flyatlas.org) and is orthologous to the mammalian AC2.^50^ Mammalian AC2 activity is Ca^2+^ independent.^51^ If ACXD is also Ca^2+^ independent, it would provide a mechanism for the engagement of distinct cAMR pathways by dDA1 for state-dependent versus experience-dependent olfactory behavior. Thus, common neural circuitry is employed for both state-dependent and conditioned behaviors with the unique changes of MBn output influenced by the intracellular signaling pathways that are mobilized.

Dopaminergic input to the dDA1 DA receptor expressed in the γ2 compartment of MBn activates an intracellular signaling pathway that includes the ACXD adenylyl cyclase, RKA activity, and the release of ACh. The downstream MBON-γ2α′1 responds to the MB ACh release through the α2 nACh receptor, with the activity of the MBON-γ2α′1 ultimately dictating the balance in state-dependent odor approach/avoidance. The simplest model to account for the state-dependent MBON activity would have the internal state modulating DA input into to the MBn to increase or decrease ACh release onto the MBON. However, our data failed to detect a significant change in ACh release between the fed and starved conditions (Figure S9B). Nevertheless, the activity of the PPL1-γ2α′1 that influences the MB γ2 compartment is required for state-dependent behavioral responses to odor (Figure 7A). Our proposed model for reconciling these observations envisions that the basal activity of this circuit is required for behavioral-state odor choice but that starvation mobilizes a qualitative or quantitative signal independent of the magnitude ACh release by the MBn to increase MBON activity. An unidentified signal representing hunger could directly enhance MBON excitability. For example, octopamine has been proposed as a feeding signal that acts directly on MBONs.^19^ Similarly, a hunger signal could act on neurons elsewhere in the brain that ultimately connect to MBONs through intermediary neurons. The hunger-responsive neuropeptide leucokinin acts on DAns that connect to MBONs.^52^ Alternatively, there may be a co-neurotransmitter released by the MBn due to starvation that works to increase MBON activity. Finally, we leave open the possibility that starvation does modulate MBn ACh output, but the reporters employed lack the sensitivity to detect this change. Future investigations into state-dependent changes in MBON-γ2α′1 physiology will need to grapple with numerous competing hypotheses.

Changes in odor responses in MBONs have suggested that learning induces a change in connectivity in the MBn-MBON synapse.^25,31,53,54^ In addition, compartment specific plasticity in MB ACh release was discovered that fits with the idea that plasticity observed in MBONs occurs from the input of MB compartments.^30,31^ However, there has been a lack of data connecting the MBn ACh release plasticity with MBON plasticity and particularly to the central role for the rut adenylyl cyclase. Our data offer this important connection. We show that the MB γ2 compartment undergoes a rapid depression in response to odor/shock pairings during aversive learning and that rut is required for the acquisition of this depression (Figures 5G–5H″). Downstream of the MB γ2 compartment, MBON-γ2α′1 drives approach^49^ and also undergoes a learning-induced depression.^34^ These results put prior speculation about how the genetic regulation of cAMP signaling through the rut adenylyl cyclase drives *Drosophila* memory on concrete ground. Our work does not conclusively delineate a role for dDA1 in the MB plasticity. We found that loss of MB dDA1 dramatically reduced naive odor responses in MB γ2 (Figure 3A). This precluded attempts to measure dDA1 effects on MB *γ2* depression because the naive responses were already low. Interestingly, both learning- and starvation-dependent odor avoidance require PKA. A likely explanation is that rut and ACXD are spatially segregated, creating distinct cAMP microdomains or signaling platforms.^55^ Thus, PKA activity would result in the phosphorylation of unique substrates within those microdomains.

The characterization of the MB as a brain region for learned, but not innate, olfactory behavior was motivated by experiments eliminating MBn or blocking MB output. Disrupting the MB eliminates odor-associated memory but has no effect on innate avoidance of those same odors.^56–58^ Recent work has overturned this simple categorization demonstrating some DANs, MBONs, and MBns do contribute to innate olfactory behavior in certain circumstances.^12,19–21,40–42,59–61^ Interestingly, the majority of reports define a role for MBns in innate behavioral responses to food-related odors.^12,18–20,40–42^ Our results, using more general, non-food odors, puts the hypothesis that MBn regulates innate behavior on more solid ground. Importantly, we found that MB dDA1 is required for state-dependent behavior to general odors (Figure 5C). This is in contrast to a report finding that dDA1 is not involved but that DAMB is required for state-dependent behavior to food odors.^20^ This difference will be a key element to understand state-dependent behavior moving forward.

The *Drosophila* and mammalian olfactory systems are remarkably similar in terms of anatomical organization and function.^62^ In both, odorant molecules activate olfactory sensory neurons (OSNs), with each OSN only expressing one type of odorant receptor (OR).^63^ Each OSN expressing the same type of OR project to the same glomeruli.^63,64^ Within the glomeruli, the OSNs synapse onto projection neuron (PN) dendrites,^64–66^ and PN activity is modified in the glomeruli by local inhibitory interneurons before being sent on to multiple higher-order brain regions.^63,67^ PN neurons connect to downstream neurons in the mammalian piriform cortex^68^ and in the *Drosophila* MB^69^ in a seemingly random manner. Like the *Drosophila* MB, the piriform cortex is critical for olfactory memory.^17,68,70,71^ It is not clear how the piriform cortex is involved in state-dependent olfactory behavior. However, in humans, odor coding changes in the piriform cortex with hunger^72^ and sleep deprivation,^73^ and piriform cortex neuron activity levels are inversely correlated with sexual satiety in rats.^74^

### Limitations of the study

We conclude from our results demonstrating dDA1-dependent MBn Ach release and a dDA1-dependent MBON-γ2α′1 Ca^2+^ in response to odor that dDA1 directly modulates the MBn/MBON connection. However, due to a limitation in the sensitivity of the ACh sensor employed, we were unable to directly record ACh input to MBON-γ2α′1. Based on the established direct cholinergic connection between these MBn and MBONs^21^ and the lack of any known non-MB cholinergic innervation to this brain region, we believe that our conclusions are merited. However, we must leave open as a formal possibility that other intermediary neurons mediate this relationship.

## STAR★METHODS

### RESOURCE AVAILABILITY

#### Lead contact

Requests for further information, resources, and reagents should be directed to the lead contact, Ronald Davis (ronalddavis@ufl.edu).

#### Materials availability

This study did not generate new unique materials.

#### Data and code availability

- All data are provided in the figures and supplemental figures.
- This paper does not report original code.
- Any additional information is available from the lead contact upon request.

### EXPERIMENTAL MODEL AND SUBJECT DETAILS

#### Husbandry

*Drosophila* stocks were maintained at room temperature and raised on standard food. Experimental crosses were maintained with a 12-h light/12 h dark cycle at approximately 70% humidity and at 25°C. Gal4 control flies did not contain the UAS element and UAS control flies did not contain gal4 element. For behavior, a mix of male and female flies were used. For physiological experiments, only female flies were used. *Drosophila* genotypes are listed in the Key Resource table.

### METHOD DETAILS

#### *In vivo* imaging and odor delivery

*In vivo* imaging was performed on 3–5 day old flies as previously described.^34^ First, the fly proboscis was immobilized with melted myristic acid. The fly was then placed into the imaging platform such that a portion of the head was in a hole in the platform. The portion of the head to be dissected was located above the platform and the rest of the body including antennae below the platform. Any gaps between the hole and the head were sealed with UV curing glue. With the top of the head submerged in saline (124 mM NaCl, 3 mM KCl, 20 mM MOPS, 1.5 mM CaCl2, 4 mM MgCl2, 6H2O, 5 mM NaHCO3, 1 mM NaH2PO4, H2O, 10 mM trehalose, 7 mM sucrose, 10 mM glucose, pH 7.2), the cuticle above the brain was removed and any fat and trachea was removed. Imaging experiments employing starved flies and control (fed) flies employed saline without the three sugars. The platform was positioned under the 25x water immersion objective of the confocal microscope. All imaging experiments were performed on a Leica TCS SP5 II or Leica TCS SP8 confocal microscope. Recordings were made with 256 × 256 resolution at 5 Hz and a 488 nm excitation. This was paired with a bandpass emission filter of 500–550 nm for GFP-based sensors and 600–700nm for tdtomato and fusionred.

Odor delivery to the immobilized flies was performed as previously described.^34^ A constant airflow of 1100 mL/min was directed at the fly′s antenna; 1000 mL/min of room air and 100 mL/min through a vial of pure mineral oil. For odor delivery, a solenoid redirected the 100 mL/min airflow through a vial of odorant diluted in mineral oil for 1 s. MCH and OCT were diluted 1:500 in mineral oil and BENZ and PA were diluted 1:1000 in mineral oil. All flies were maintained on normal fly food until recording unless otherwise stated. Starved flies were moved to vials without food but with a kimwipe soaked with 4 mL of deionized water 28–30 h prior to testing.

ROIs were drawn individually around the γ2–5 compartments based on previous anatomical characterizations and similar experimental designs.^22,25,30^ For odor response traces using GRAB-ACh and GCaMP, a baseline was calculated using the mean fluorescence during the 5 s prior to each odor presentation. ΔF/F_0_ was calculated using this baseline. Average odor responses were calculated by taking the mean ΔF/F_0_ for the 2s beginning at odor delivery onset. Ongoing DAN Ca^2+^ activity was calculated by first dividing the GCaMP signal to the fusionred signal at each frame. Next, ΔF/F_0_ was calculated using a 100 frame moving baseline. Activity per second was calculated by dividing the mean DF/F_0_ across the whole recording by seconds of the recording. Dopamine input to MBn in Figures 1C′ and 1C″ were calculated by first dividing the GRAB-DA signal by the tdtomato signal at each frame. For Figure 1C′ ΔF/F_0_ was calculated by setting a baseline using the 30 s prior to BAPTA-AM application. Dopamine input for Figures 1D′ and 1D″ was similarly calculated except there was no normalization to tdtomato, as it was not expressed.

#### *In vivo* imaging during aversive conditioning

Flies were prepared and odor was delivered as described in the previous section. A copper grid was positioned below the fly so that all 6 legs were contacting the grid. This grid was used to deliver 1.25 s of 90V electric shock during training. Priorto training (Pre) and 2 min after training (Post), recordings were made of odor responses to MCH, OCT, and BENZ. The flies were trained with 3 cycles of the training protocol. One cycle of training consisted of the following: CS+ (OCT) odor pulses were 2 s long. After 0.75 s of each odor pulse, a 1.25 s of 90V was delivered to the fly such that the odor and electric shock co-terminated. Four of these CS+/shock pairings were given with 5 s between pairings. Thirty seconds after the last CS+/shock pairing, 4 CS- (MCH) odor pulses were given, 5 s apart with each pulse being 2 s long. There was 1 min between training cycles. GRAB-ACh odor response traces were generated as described above except for recordings during training. In this case, baselines were set before the first odor delivery in each training cycle and used for each of the three additional odor deliveries in the cycle. Maximum odor responses were calculated by taking the maximum ΔF/F_0_ for the 2 s beginning at odor delivery onset.

#### In vivo and ex *vivo* bath application

All solutions were perfused in at the indicated times at a flow rate of 2 mL/min. BAPTA-AM (Sigma/Millipore A1076) was used at a final concentration of 50 μM in Ca^2^+/Mγ2+-free saline. Forskolin (Sigma F6886) was used at a final concentration of 100 mM in saline. KCl was used at a final concentration of 100 μM in saline.

#### Innate avoidance

Fed flies were maintained on normal fly food until testing. Starved flies were moved to vials without food but with a kimwipe soaked with 4 mL of deionized water 28–30 h prior to testing. Flies were transferred to a T-maze, allowing 2 min for flies to distribute between the 2 arms, one delivering the odor (diluted in mineral oil) to be tested and the other delivering no odor (mineral oil only). A PI was calculated for each group using the formula (number of flies in the odor arm) - (number of flies in non-odor arm)/total flies in both arms. Starved-Fed odor avoidance scores were calculated by subtracting the Fed odor avoidance score from the Starved odor avoidance score.

#### Aversive conditioning

Aversive olfactory conditioning experiments were performed on 1–5 day old flies as previously described.^75^ Training and testing was performed in a dark room lit with red light with atmospheric conditions of 25°C and approximately 70% humidity. A group of 55–60 flies were placed into training tubes and subjected to the aversive learning protocol: 30 s of air, followed by 1 min of the CS+ odor and simultaneous electric shock, then 30 s of air, followed by 1 min of the CS-odor, then 30 s of air. Electric shock was delivered in 12 pulses at 90 V during the 1-min period. The odors used were 4-methylcyclohexonal (MCH) and 3-octanol (OCT) diluted in mineral oil. Flies were transferred to a T-maze, allowing 2 min for flies to distribute between the 2 arms, one carrying the CS+ and the other carrying the CS-air stream. One group was trained to the CS+ MCH and CS-OCT and the other to CS+ OCT and CS-MCH. A half PI was calculated for each group using the formula (number of flies in CS-arm)-(number of flies in CS+ arm)/total flies in both arms. The two-half PIs for the two mazes were averaged to produce the final PI.

### QUANTIFICATION AND STATISTICAL ANALYSIS

#### Quantification and statistics

The statistics used are described in the corresponding Figure legends. Statistical analyses were performed using Prism 5.0.

## Supplementary Material

1

## Figures and Tables

**Table:** KEY RESOURCES TABLE

REAGENT or RESOURCE	SOURCE	IDENTIFIER
Chemicals, peptides, and recombinant proteins		

BAPTA-AM	Sigma/Millipore	Cat# A1076
Forskolin	Sigma	Cat# F6886

Experimental models: Organisms/strains		

*D.melanogaster: tdtomato,* w[*]; P{y[+t7.7] w[+mC] = 10XUAS-IVS-myr::tdTomato}attP2	BDSC	RRID:BDSC_32221
*D.melanogaster: R1SF02-gal4,* w[1118]; P{y[+t7.7] w[+mC] = GMR13F02GAL4}attP2	BDSC	RRID:BDSC_48571
*D.melanogaster: TH-gal4,* w[*]; P{w[+mC] = ple-GAL4.F3	BDSC	RRID:BDSC_8848
*D.melanogaster*: *R58E02-gal4*, w[1118]; P{y[+t7.7] w[+mC] = GMR58E02-GAL4}attP2	BDSC	RRID:BDSC_41347
*D.melanogaster: TNT*, w[*]; P{w[+mC] = UAS-TeTxLC.tnt}Γ2	BDSC	RRID:BDSC_28838
*D.melanogaster: NaChBac*, y[1] w[*]; P{w[+mC] = UAS-NaChBac}2	BDSC	RRID:BDSC_9469
*D.melanogaster*: *DAMB RNAi*, y[1] sc[*] v[1] sev[21]; P{y[+t7.7] v[+t1.8] = TRiP.HMC′2893}attP2	BDSC	RRID:BDSC_51423
*D.melanogaster*: *DOP2R RNAi*, y[1] v[1]; P{y[+t7.7] v[+t1.8] = TRiP.JF02025} attP2y[1] v[1]; P{y[+t7.7] v[+t1.8] = TRiP.JF02025}attP2	BDSC	RRID:BDSC_26001
*D.melanogaster: dDA1 RNAi-1*, y[1] v[1]; P{y[+t7.7] v[+t1.8] = TRiP.HM04077}attP2	BDSC	RRID:BDSC_31765
*D.melanogaster: dDA1 RNAi-2*, P{KK102341}VIE-260B	VDRC	FBst0478881
*D.melanogaster: DUMB2*, PBac{WH}Dop1R1f02676	VDRC	FBal0184074
*D.melanogaster*: *Syt-GCaMP*, w[*]; P{y[+t7.7] w[+mC] = UAS-sytGCaMP6s} attP40;	BDSC	RRID:BDSC_64415
*D.melanogaster: Control (S6S0S)*, y[1] v[1]; P{y[+t7.7] = CaryP}attP2	BDSC	RRID:BDSC_36303
*D.melanogaster*: *Dunce RNAi*, y[1] v[1]; P{y[+t7.7] v[+t1.8] = TRiP.JF02561} attP2	BDSC	RRID:BDSC_27250
*D.melanogaster*: *Rutabaga RNAi-1*, y[1] v[1]; P{y[+t7.7] v[+t1.8] = TRiP.JF02361} attP2	BDSC	RRID:BDSC_27035
*D.melanogaster: Rutabaga RNAi-2*, P{KK109441}VIE-260B	VDRC	FBst0473632
*D.melanogaster: Control (60100)*, y,w[1118]; P{attP,y[+],w[3′]	VDRC	60100
*D.melanogaster: Control (60000)*, w[1118]	VDRC	60000
*D.melanogaster: Control (S6S04)*, y[1] v[1]; P{y[+t7.7] = CaryP}attP40	BDSC	RRID:BDSC_36304
*D.melanogaster. GCaMP6m-FusionRed* (Gusion),	Greg Macleod	N/A
*D.melanogaster. GRAB-DA (UAS)*, w[*]; P{y[+t7.7] w[+mC] = 10XUAS-GRAB(DA2m)} attP40	BDSC	RRID.BDSC_90878
*D.melanogaster. GRAB-DA (lexA)*, w[*]; P{y[+t7.7] w[+mC] = 10XlexAop-GRAB(DA2m)} attP40	BDSC	RRID.BDSC_90879
*D.melanogaster. GRAB-Ach* *(UAS, 2ND Chromosome)*, w[*]; P{y[+t7.7] w[+mC] = 10XUAS-GRAB(ACh3.0)}attP40	BDSC	RRID.BDSC_86549
*D.melanogaster*. *GRAB-Ach (UAS, 3rd Chromosome)*, w[*]; PBac{y[+mDint2] w[+mC] = 10XUAS-GRAB (ACh3.0)}VK00005	BDSC	RRID.BDSC_86550
*D.melanogaster*. *GRAB-Ach* *(lexAop, 2ND Chromosome)*, w[*]; P{y[+t7.7] w[+mC] = 10XlexAop-GRAB (ACh3.0)}attP40	BDSC	RRID.BDSC_86551
*D.melanogaster. GRAB-Ach (lexAop, 3rd Chromosome)*, w[*]; PBac{y[+mDint2] w[+mC] = 10XlexAop-GRAB (ACh3.0)}VK00005	BDSC	RRID.BDSC_86552
*D.melanogaster. Ac78C*, y[1] sc[*] v[1] sev[21]; P{y[+t7.7] v[+t1.8] = TRiP.HMC′3999}attP2	BDSC	RRID.BDSC_55312
*D.melanogaster. ACXD*, y[1] sc[*] v[1] sev[21]; P{y[+t7.7] v[+t1.8] = TRiP.GL00425}attP2	BDSC	RRID.BDSC_35589
*D.melanogaster. Pka-C2*, y[1] v[1]; P{y[+t7.7] v[+t1.8] = TRiP.JF01756}attP2	BDSC	RRID.BDSC_31243
*D.melanogaster. CrebB*, y[1] v[1]; P{y[+t7.7] v[+t1.8] = TRiP.JF02494} attP2	BDSC	RRID.BDSC_29332
*D.melanogaster. Cngl*, y[1] v[1]; P{y[+t7.7] v[+t1.8] = TRiP.JF03099} attP2	BDSC	RRID.BDSC_28684
*D.melanogaster. Pka-C2*, y[1] v[1]; P{y[+t7.7] v[+t1.8] = TRiP.JF01448} attP2	BDSC	RRID.BDSC_31656
*D.melanogaster. CrebA*, y[1] v[1]; P{y[+t7.7] v[+t1.8] = TRiP.JF02823}attP2	BDSC	RRID.BDSC_27648
*D.melanogaster. Pka-C3*, y[1] v[1]; P{y[+t7.7] v[+t1.8] = TRiP.JF02723}attP2	BDSC	RRID.BDSC_27569
*D.melanogaster. Epac*, y[1] v[1]; P{y[+t7.7] v[+t1.8] = TRiP.JF02476}attP2	BDSC	RRID.BDSC_29317
*D.melanogaster. Pka-C1 RNAi-1*, y[1] v[1]; P{y[+t7.7] v[+t1.8] = TRiP.JF01218}attP2	BDSC	RRID.BDSC_31277
*D.melanogaster. Pka-R1*, y[1] v[1]; P{y[+t7.7] v[+t1.8] = TRiP.JF02788}attP2	BDSC	RRID.BDSC_27708
*D.melanogaster. CngA*, y[1] v[1]; P{y[+t7.7] v[+t1.8] = TRiP.JF02039}attP2	BDSC	RRID.BDSC_26014
*D.melanogaster. CngB*, y[1] v[1]; P{y[+t7.7] v[+t1.8] = TRiP.JF02034}attP2	BDSC	RRID.BDSC_26009
*D.melanogaster*: *Pka-C1 RNAi-2*, y[1] v[1]; P{y[+t7.7] v[+t1.8] = TRiP.JF01188}attP2	BDSC	RRID:BDSC_31599
*D.melanogaster. Pka-R2 RNAi-1*, y[1] v[1]; P{y[+t7.7] v[+t1.8] = TRiP.JF02759}attP2	BDSC	RRID:BDSC_27680
*D.melanogaster: CrebA*, y[1] v[1]; P{y[+t7.7] v[+t1.8] = TRiP.JF02189}attP2	BDSC	RRID:BDSC_31900
*D.melanogaster: AC3*, y[1] v[1]; P{y[+t7.7] v[+t1.8] = TRiP.JF03041}attP2	BDSC	RRID:BDSC_28626
*D.melanogaster: Akap200*, y[1] v[1]; P{y[+t7.7] v[+t1.8] = TRiP.HM05018}attP2	BDSC	RRID:BDSC_28532
*D.melanogaster: CngB*, y[1] v[1]; P{y[+t7.7] v[+t1.8] = TRiP.HMJ21964}attP40	BDSC	RRID:BDSC_58072
*D.melanogaster: ACXC*, y[1] sc[*] v[1] sev[21]; P{y[+t7.7] v[+t1.8] = TRiP.HMC′6401} attP40	BDSC	RRID:BDSC_67298
*D.melanogaster: ACXB*, y[1] sc[*] v[1] sev[21]; P{y[+t7.7] v[+t1.8] = TRiP.HMC′5250}attP40	BDSC	RRID:BDSC_62243
*D.melanogaster: CΓ43373*, y[1] sc[*] v[1] sev[21]; P{y[+t7.7] v[+t1.8] = TRiP.HMC′5757}attP4	BDSC	RRID:BDSC_64884
*D.melanogaster: Ac13E*, y[1] sc[*] v[1] sev[21]; P{y[+t7.7] v[+t1.8] = TRiP.HMC′5254}attP40	BDSC	RRID:BDSC_62247
*D.melanogaster: ACXD*, y[1] sc[*] v[1] sev[21]; P{y[+t7.7] v[+t1.8] = TRiP.HMC′5344} attP40	BDSC	RRID:BDSC_62871
*D.melanogaster: CΓ32301*, y[1] sc[*] v[1] sev[21]; P{y[+t7.7] v[+t1.8] = TRiP.HMC′5252}attP40	BDSC	RRID:BDSC_62245
*D.melanogaster: CΓ32305*, y[1] sc[*] v[1] sev[21]; P{y[+t7.7] v[+t1.8] = TRiP.HMC′5253}attP40	BDSC	RRID:BDSC_62246
*D.melanogaster: ACXA*, y[1] sc[*] v[1] sev[21]; P{y[+t7.7] v[+t1.8] = TRiP.HMC′5251}attP40	BDSC	RRID:BDSC_62244
*D.melanogaster: CrebB*, y[1] v[1]; P{y[+t7.7] v[+t1.8] = TRiP.HMJ30249} attP40/Cy0	BDSC	RRID:BDSC_63681
*D.melanogaster: Pka-R2 RNAi-1*, y[1] v[1]; P{y[+t7.7] v[+t1.8] = TRiP.HMJ21276} attP40	BDSC	RRID:BDSC_53930
*D.melanogaster: Ac3*, y[1] v[1]; P{y[+t7.7] v[+t1.8] = TRiP.HMJ30182} attP40	BDSC	RRID:BDSC_63615
*D.melanogaster: CrebA*, y[1] v[1]; P{y[+t7.7] v[+t1.8] = TRiP.HMJ02218} attP40	BDSC	RRID:BDSC_42562
*D.melanogaster*: *CΓ32301*, y[1] v[1]; P{y[+t7.7] v[+t1.8] = TRiP.HMJ21904} attP40	BDSC	RRID:BDSC_57857
*D.melanogaster: CΓ43373*, P{KK110535}VIE-260B	VDRC VDRC	FBst0473929
*D.melanogaster: Acxc*, FBst0476995 P{KK103168}VIE-260B	VDRC	FBst0476995
*D.melanogaster: ACXB/AC34A*, P{KK106308}VIE-260B	VDRC	FBst0476106
*D.melanogaster: Ac76E*, P{KK105800}VIE-260B	VDRC	FBst0478057
*D.melanogaster: ACXD*, P{KK106797}VIE-260B	VDRC	FBst0479218
*D.melanogaster: CΓ32305*, P{KK106912}VIE-260B	VDRC	FBst0472976
*D.melanogaster: CΓ32305*, w[1118]; P{GD14845}v36592	VDRC	FBst0461757
*D.melanogaster: ACXA*, w[1118]; P{GD16283}v49100	VDRC	FBst0468273
*D.melanogaster: CΓ43373*, w[1118]; P{GD13449}v23385	VDRC	FBst0454980
*D.melanogaster: Acxc*, w[1118]; P{GD1108}v2870	VDRC	FBst0457608
*D.melanogaster: CΓ32301*, w[1118]; P{GD3573}v12238	VDRC	FBst0450451
*D.melanogaster: Ac13E*, w[1118]; P{GD3374}v11547	VDRC	FBst0450290
*D.melanogaster: CΓ32301*, w[1118]; P{GD16147}v47917	VDRC	FBst0467617
*D.melanogaster: ACXB*, w[1118]; P{GD1109}v9748	VDRC	FBst0471592
*D.melanogaster: Ac7SC*, w[1118]; P{GD2764}v51979	VDRC	FBst0469649
*D.melanogaster: Ac3*, w[1118]; P{GD1799}v33217	VDRC	FBst0459989
*D.melanogaster: Ac76e*, w[1118] P{GD2737}v51974	VDRC	FBst0469645
*D.melanogaster: ACXD overexpression*, w[*]; P{w[+mC] = UAS-ACXD.D}3	BDSC	RRID:BDSC_68218
*D.melanogaster: PKA-R2 RNAi-2*, P{KK109446}VIE-260B	VDRC	FBst0473636
*D.melanogaster: 1471-gal4*, w[1118]; P{w[+mW.hs] = GawB}1471	BDSC	RRID:BDSC_9465
*D.melanogaster: THD′-gal4,;* P{TH-D′-GAL4};	BDSC	RRID:BDSC_93704
*D.melanogaster: MB296B split gal4*, w[1118]; P{y[+t7.7] w[+mC] = R15B′1-p65.AD} attP40; P{y[+t7.7] w[+mC] = R26F01-GAL4.DBD}attP2	BDSC	RRID:BDSC_68308
*D.melanogaster: MB′99C split gal4*, w[1118]; P{y[+t7.7] w[+mC] = R73F07-GAL4.DBD}attP2 PBac{y[+mDint2] w[+mC] = ple-p65.AD}VK00027	BDSC	RRID:BDSC_68290
*D.melanogaster: R25D′1-lexa*, w[1118]; P{y[+t7.7] w[+mC] = GMR25D′1-lexA}attP40	BDSC	RRID:BDSC_53519
*D.melanogaster: C305a-gal4*, w[*]; P{w[+mW.hs] = GawB}Cka[c305a]	BDSC	RRID:BDSC_30829
*D.melanogaster: MB′77C split gal4*, w[1118]; P{y[+t7.7] w[+mC] = R19F09-GAL4.DBD}attP2 PBac{y[+mDint2] w[+mC] = R25D′1-p65.AD}VK00027	BDSC	RRID:BDSC_68284
*D.melanogaster: nACHRa1 RNAi*, y[1] v[1]; P{y[+t7.7] v[+t1.8] = TRiP.JF03103}attP2	BDSC	RRID:BDSC_28688
*D.melanogaster: nACHRa2 RNAi*, y[1] v[1]; P{y[+t7.7] v[+t1.8] = TRiP.JF02643}attP2	BDSC	RRID:BDSC_27493
*D.melanogaster: nACHRa3 RNAi*, y[1] v[1]; P{y[+t7.7] v[+t1.8] = TRiP.JF02750}attP2	BDSC	RRID:BDSC_27671
*D.melanogaster: nACHRa4 RNAi*, y[1] v[1]; P{y[+t7.7] v[+t1.8] = TRiP.JF03419}attP2	BDSC	RRID:BDSC_31985
*D.melanogaster: nACHRa5 RNAi*, y[1] v[1]; P{y[+t7.7] v[+t1.8] = TRiP.JF01963}attP2	BDSC	RRID:BDSC_25943
*D.melanogaster: nACHRa6 RNAi*, y[1] v[1]; P{y[+t7.7] v[+t1.8] = TRiP.JF01853}attP2	BDSC	RRID:BDSC_25835
*D.melanogaster: nACHRa7 RNAi*, y[1] v[1]; P{y[+t7.7] v[+t1.8] = TRiP.JF02570}attP2	BDSC	RRID:BDSC_27251
*D.melanogaster: Dcr2*, w1118; P{UAS-Dicer2};	BDSC	RRID:BDSC_24650

## Highlights

- Dopamine controls the magnitude of odor-driven mushroom body (MB) neurotransmission
- MB dopamine receptor signaling through ACXD enhances odor responses, driving innate avoidance
- MB dopamine receptor signaling via rutabaga depresses odor responses, driving memory formation
- Both odor learning and innate avoidance require dopamine signaling in the MB γ2 compartment
