# Supplementary material for: Innate and learned odor-guided behaviors utilize distinct molecular signaling pathways in a shared dopaminergic circuit
Source: Cell Rep. Author manuscript; Available in PMC 2023 Oct 23. (PMC10366338; doi:10.1016/j.celrep.2023.112026)
Supplement: 1 [file NIHMS1878905-supplement-1.pdf]

Cell Reports, Volume *42*

Supplemental information

Innate and learned odor-guided behaviors utilize distinct molecular signaling pathways in a shared dopaminergic circuit

Nathaniel C. Noyes and Ronald L. Davis

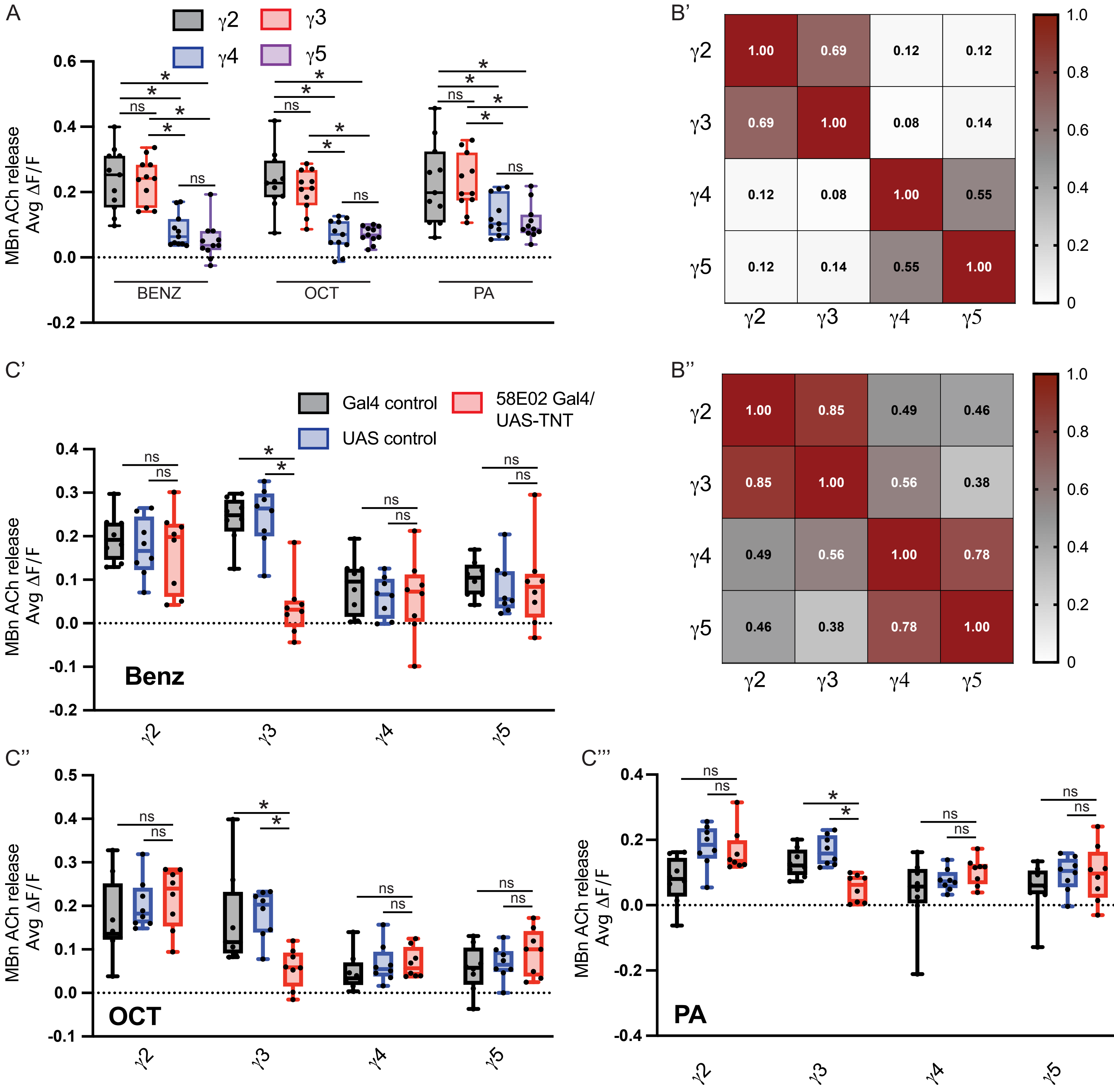
A
γ2 γ3 γ4 γ5
MBn ACh release Avg ΔF/F
BENZ
OCT
PA
ns
*
B'
B''
1.00 0.69 0.12 0.12
0.69 1.00 0.08 0.14
0.12 0.08 1.00 0.55
0.12 0.14 0.55 1.00
1.00 0.85 0.49 0.46
0.85 1.00 0.56 0.38
0.49 0.56 1.00 0.78
0.46 0.38 0.78 1.00
C'
Gal4 control
UAS control
58E02 Gal4/ UAS-TNT
Benz
C''
OCT
C'''
PA

Figure S1

Figure S1. Dopamine controls MBn ACh neurotransmission. Related to Figure 2. (A) The GRAB ACh sensor was expressed in MBn using R13F02-gal4 and responses to three different odors were measured. n=11 (B') Signal correlation performed on DANs GCaMP traces related to Figure 1b. (B'') Pearson correlation performed on average GRAB ACh odor responses related to Figure 1e. (C) The GRAB ACh sensor was expressed in MBn using R13F02-lexA and TNT was expressed in PAM DANs using R58E02 gal4. Average odor responses were quantified for each compartment in γ2-5. n=8 Box-and-whisker plots show the range of individual data points, with the interquartile spread as the box and the median as the line bisecting each box.*$P < 0.05$. (A) one-way ANOVA with Dunn's test. (C) one-way ANOVA with Kruskal-Wallis test. BENZ=benzaldehyde, OCT=3-octanol, PA= pentyl acetate.

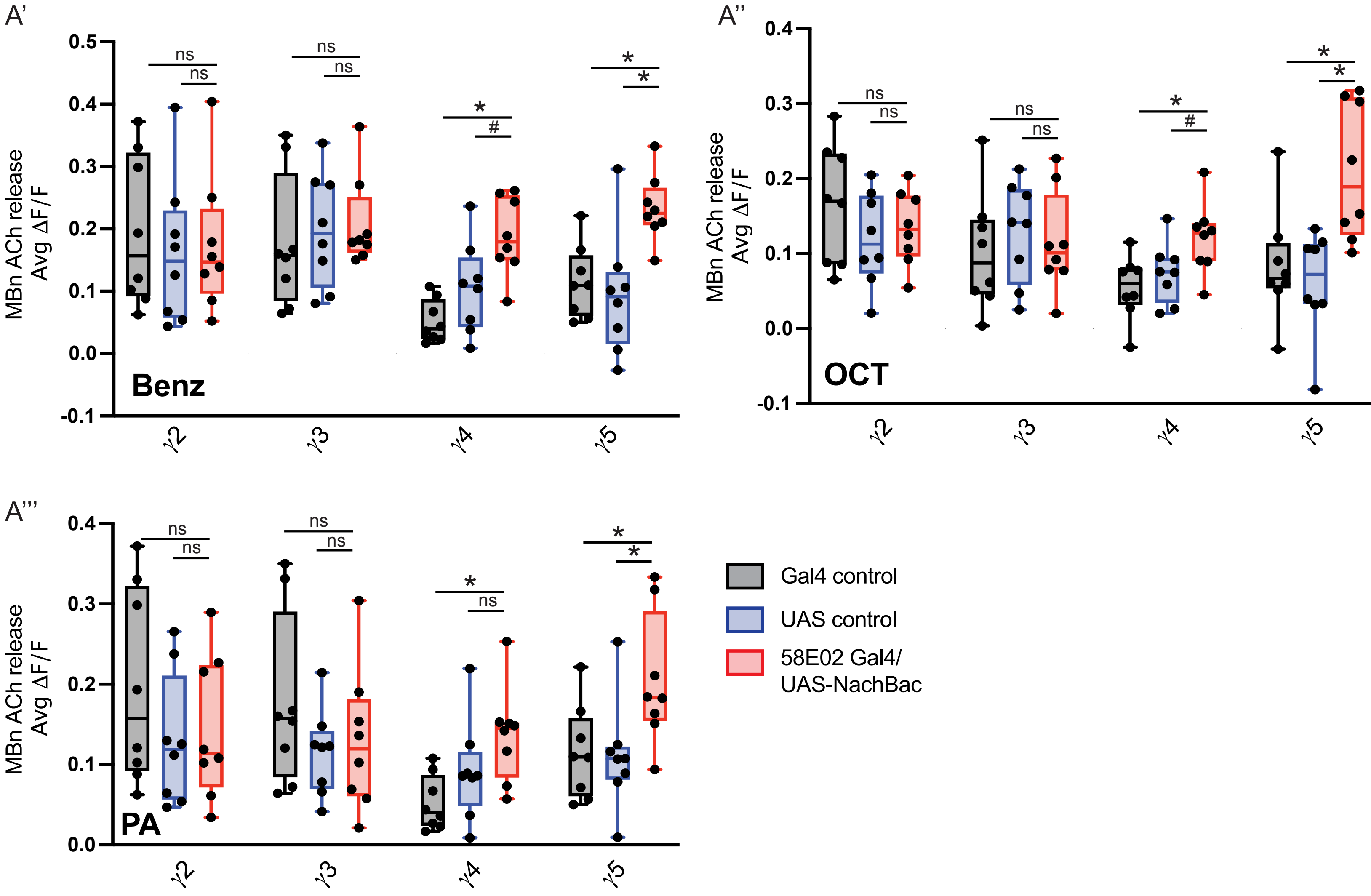
A'
MBn ACh release
Avg ΔF/F
Benz
ns
ns
*
#
*
*
γ2
γ3
γ4
γ5
A''
OCT
A'''
PA
Gal4 control
UAS control
58E02 Gal4/
UAS-NachBac

Figure S2

Figure S2. Dopamine controls MBn ACh neurotransmission. Related to Figure 2.(A) The GRAB ACh sensor was expressed in MBn using R13F02-LexA and NacBach was expressed in PAM DANs using R58E02 gal4. Average odor responses were quantified for each compartment in γ2-5. n=8 Box-and-whisker plots show the range of individual data points, with the interquartile spread as the box and the median as the line bisecting each box.*P < 0.05. (A) one-way ANOVA with Kruskal-Wallis test. BENZ=benzaldehyde, OCT=3-octanol, PA= pentyl acetate.

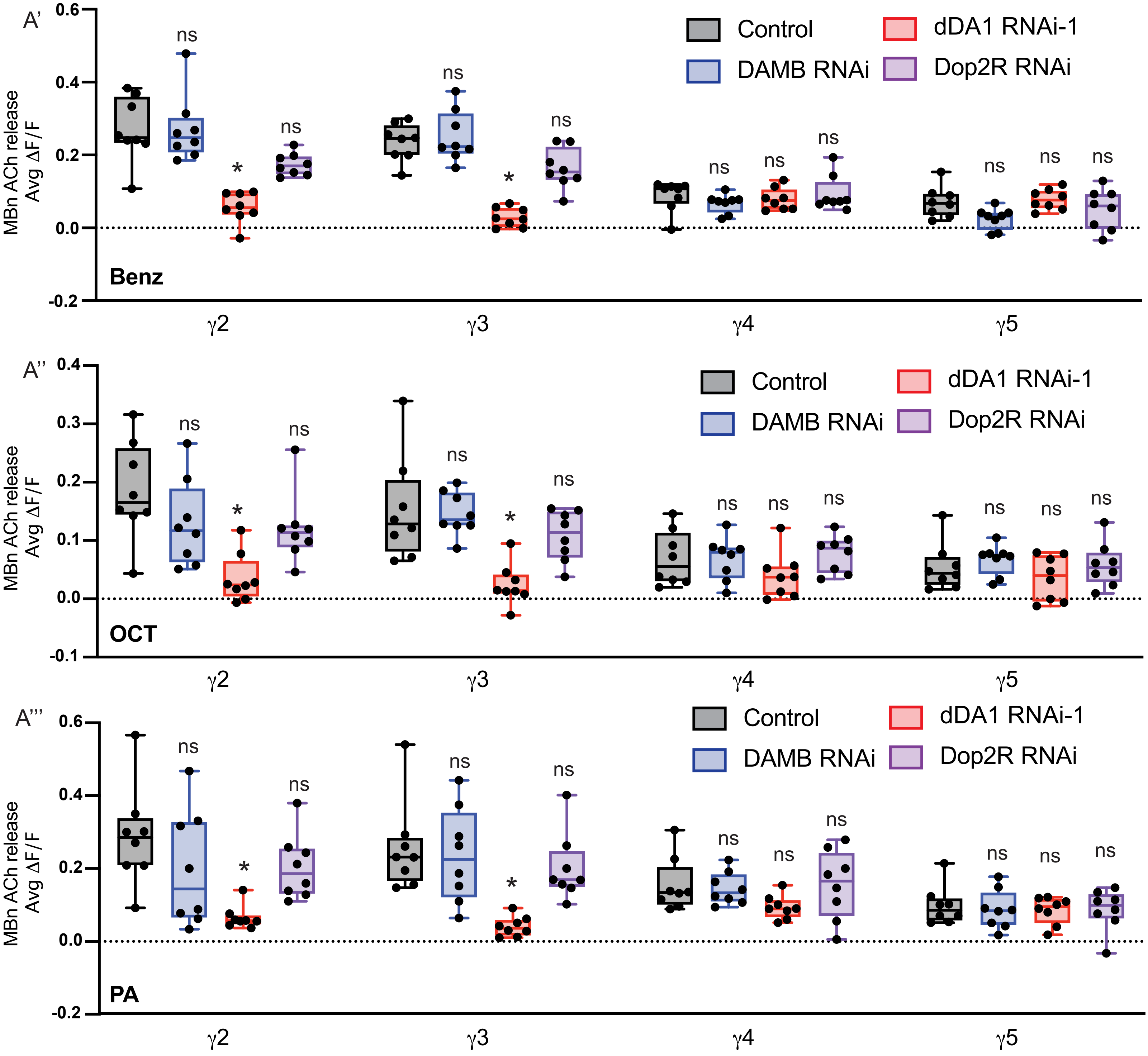

A'
Control
dDA1 RNAi-1
DAMB RNAi
Dop2R RNAi
MBn ACh release
Avg ΔF/F
Benz
γ2
γ3
γ4
γ5
A''
OCT
A'''
PA

Figure S3

Figure S3. The dDA1 dopamine receptor mediates the effects of dopamine on MB neurotransmission. Related to Figure 3. (A) The GRAB ACh sensor and RNAi transgenes were expressed in MBn using R13F02-gal4. Average odor responses were quantified for each compartment in γ2-5. n=8. Box-and-whisker plots show the range of individual data points, with the interquartile spread as the box and the median as the line bisecting each box. *P < 0.05. (A) one-way ANOVA with Kruskal-Wallis test.

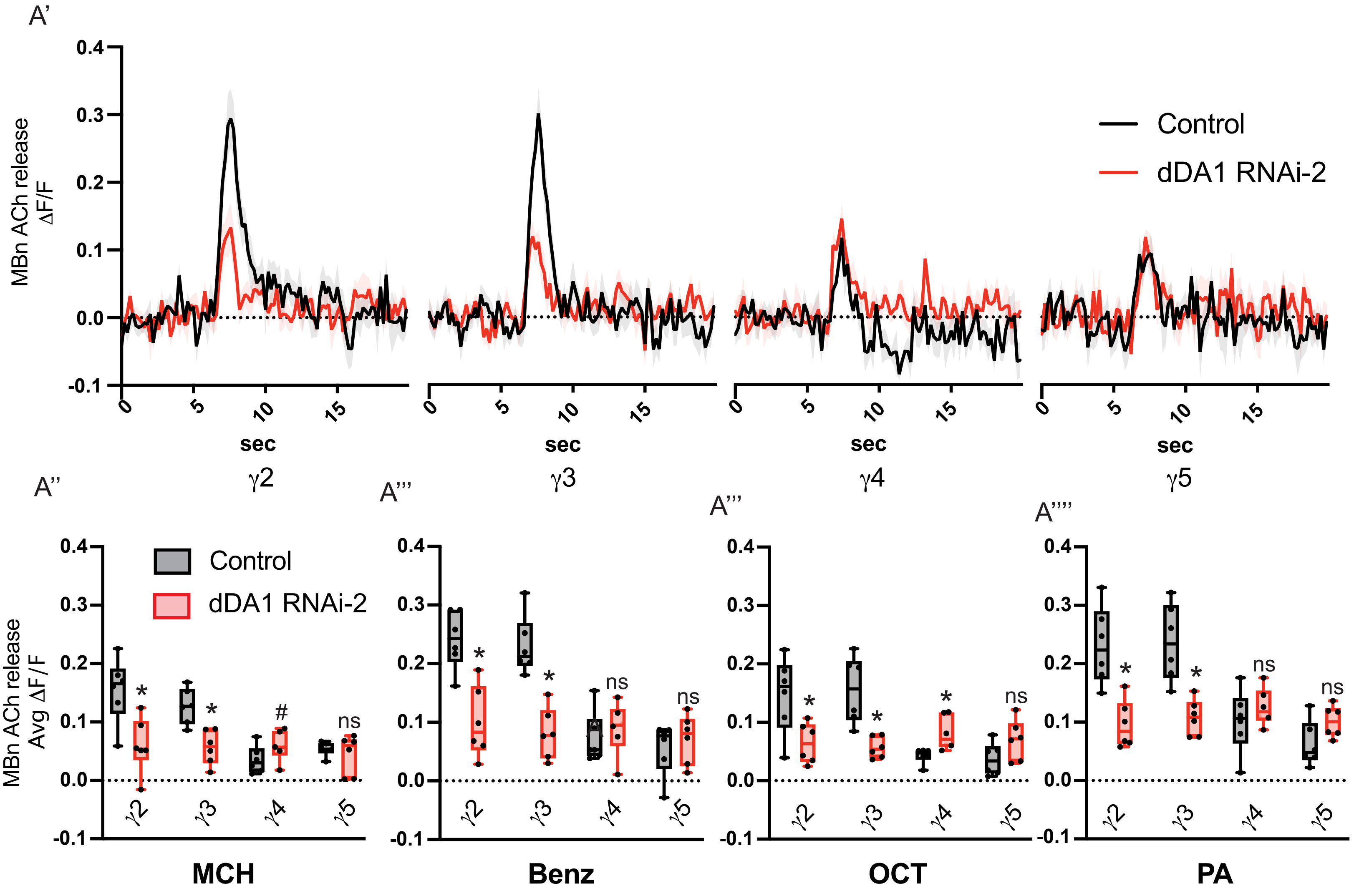

A'
A''
A'''
A'''
A''''
MBn ACh release ΔF/F
MBn ACh release Avg ΔF/F
Control
dDA1 RNAi-2
sec
γ2
γ3
γ4
γ5
MCH
Benz
OCT
PA

Figure S4

Figure S4. The dDA1 dopamine receptor mediates the effects of dopamine on MB neurotransmission. Related to Figure 3. (A) The GRAB ACh sensor and RNAi were expressed in MBn using R13F02-gal4. One second of odor (MCH) was delivered at 5-s. n=6 (A') Traces show the average response (±SEM) across all flies tested. (A''-A'''') Average odor responses were quantified for each compartment in γ2-5. Box-and-whisker plots show the range of individual data points, with the interquartile spread as the box and the median as the line bisecting each box. *$P < 0.05$. (A) Mann-Whitney test.

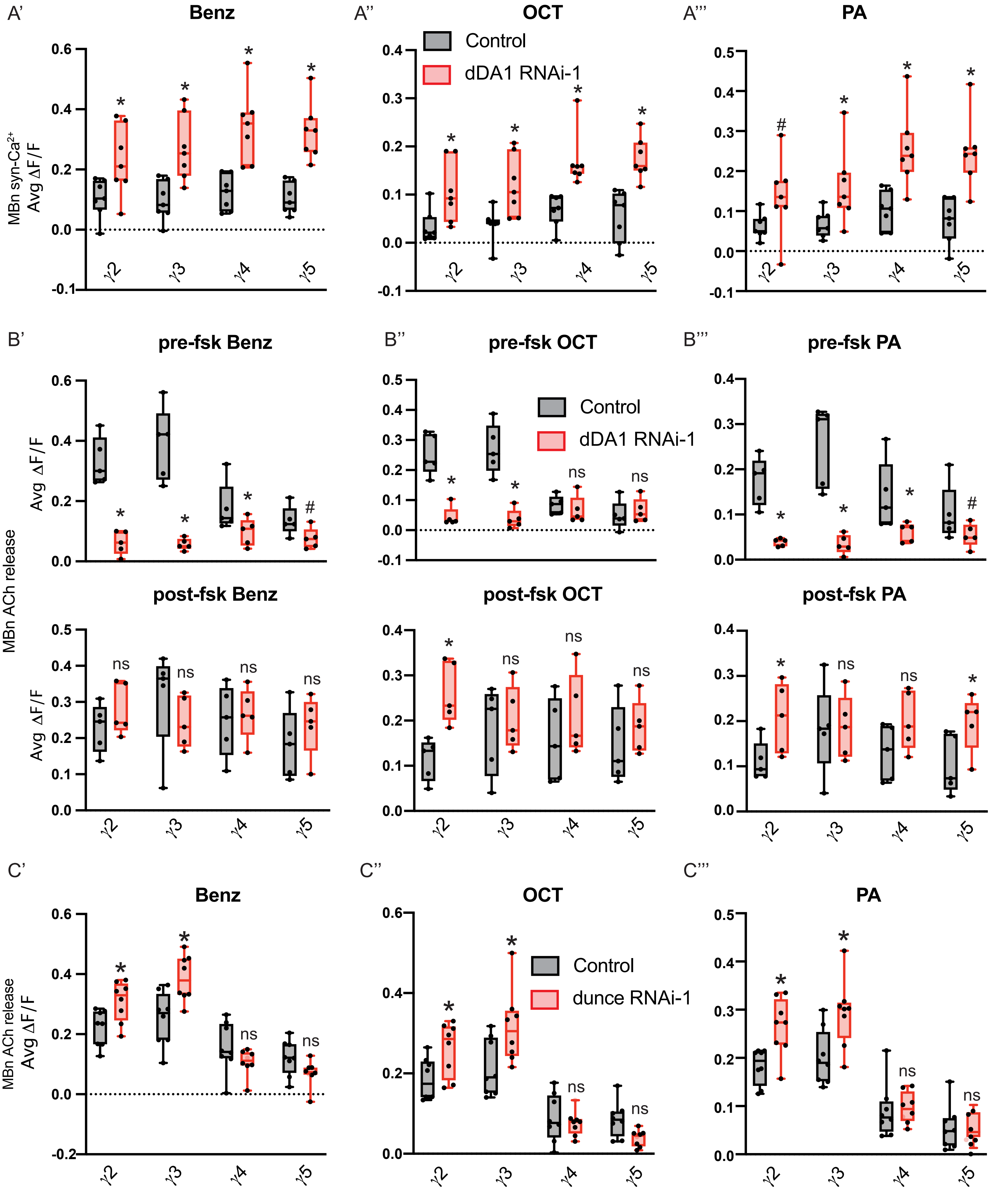

A'
Benz
A''
OCT
A'''
PA
Control
dDA1 RNAi-1
MBn syn-Ca2+
Avg ΔF/F
B'
pre-fsk Benz
B''
pre-fsk OCT
B'''
pre-fsk PA
MBn ACh release
Avg ΔF/F
post-fsk Benz
post-fsk OCT
post-fsk PA
C'
Benz
C''
OCT
C'''
PA
dunce RNAi-1
γ2
γ3
γ4
γ5
ns

Figure S5

Figure S5. cAMP mediates the effects of dopamine on MBn neurotransmission. Related to Figure 4. (A) The syt-GCaMP sensor and RNAi transgenes were expressed in MBn using R13F02-gal4. Average odor responses were quantified for each compartment in γ2-5. n=7 (B) The GRAB ACh sensor and RNAi were expressed in MBn using R13F02-gal4. Brains were dissected and imaged ex vivo in physiological saline. 50 uM forskolin was bath applied between the Pre and Post odor response tests. Average odor responses were quantified for each compartment in γ2-5 before and after forskolin application. n=5 (C) The GRAB ACh sensor and RNAi were expressed in MBn using R13F02-gal4. Average odor responses were quantified for each compartment in γ2-5. Box-and-whisker plots show the range of individual data points, with the interquartile spread as the box and the median as the line bisecting each box. n=8 *P < 0.05. (A-C) Mann-Whitney test.

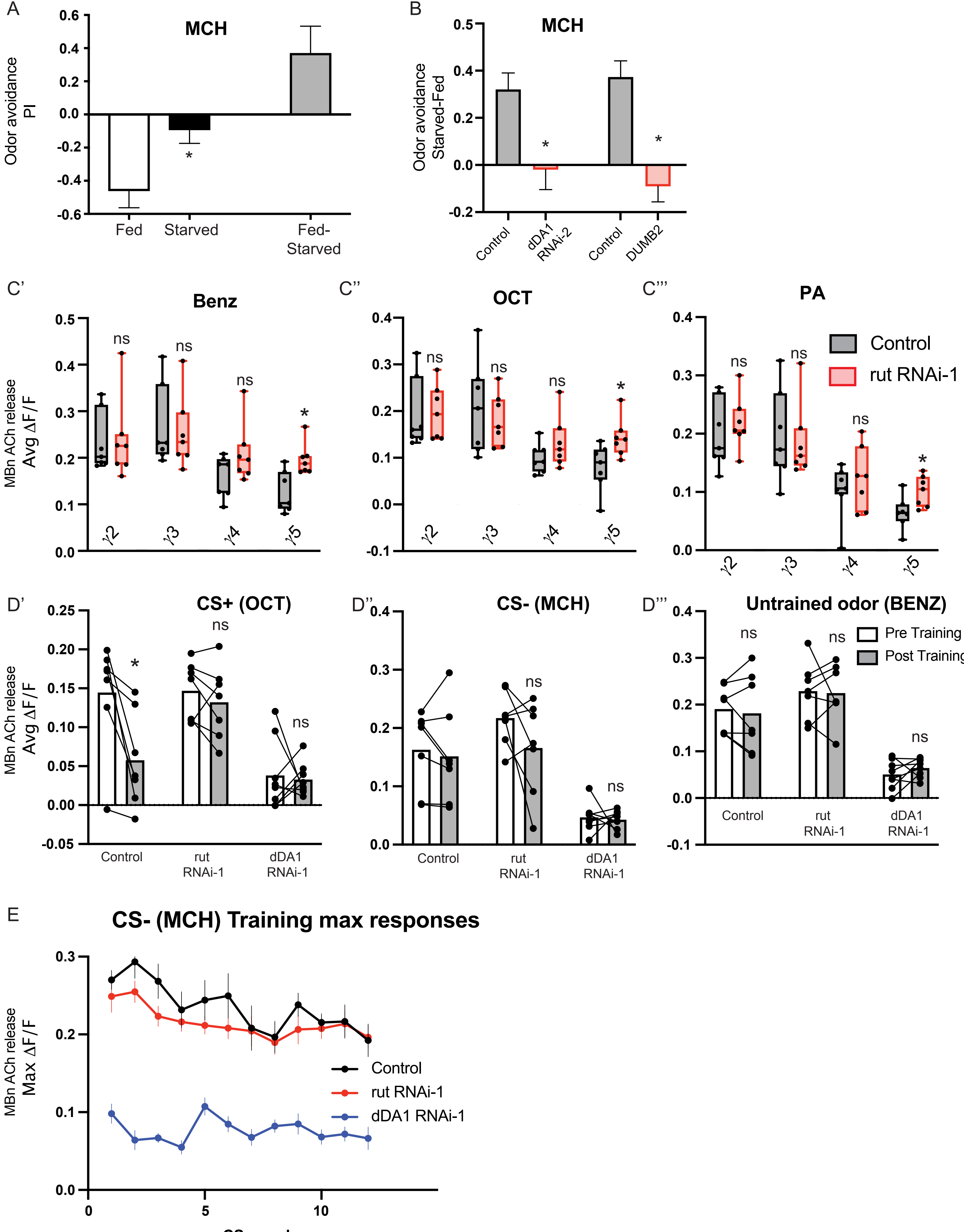
A
MCH
Odor avoidance PI
Fed
Starved
Fed-Starved
B
MCH
Odor avoidance Starved-Fed
Control
dDA1 RNAi-2
Control
DUMB2
C'
Benz
C''
OCT
C'''
PA
MBn ACh release Avg ΔF/F
Control
rut RNAi-1
ns
*
γ2
γ3
γ4
γ5
D'
CS+ (OCT)
D''
CS- (MCH)
D'''
Untrained odor (BENZ)
Pre Training
Post Training
Control
rut RNAi-1
dDA1 RNAi-1
E
CS- (MCH) Training max responses
MBn ACh release Max ΔF/F
Control
rut RNAi-1
dDA1 RNAi-1
CS- number

Figure S6

Figure S6. dDA1 controls state-dependent odor preferences and MB neurotransmission independent from rutabaga. Related to Figure 5. (A) Starved-Fed odor avoidance scores were calculated by subtracting the Fed odor avoidance score from the Starved odor avoidance score. n=8 (B) Starved-Fed odor (MCH) avoidance scores for dDA1 knockdown and dDA1 null mutant flies (DUMB2). RNAi was expressed in MBn using R13F02-gal4. n=8-10 (C) The GRAB ACh sensor and rut RNAi were expressed in MBn using R13F02-gal4. Average odor responses were quantified for each compartment in γ2-5. n=7 (D) The GRAB ACh sensor and RNAi were expressed in MBn using R13F02-gal4. Average odor responses to MCH were quantified for γ2 before and after training. n=7 (E) Maximum odor responses were quantified for each CS- odor (MCH) pulse in γ2. The maximum odor responses to the CS+ are shown in Figure 5H''. Box-and-whisker plots show the range of individual data points, with the interquartile spread as the box and the median as the line bisecting each box. *$P < 0.05$. (B-D) Mann-Whitney test. (E) one-way ANOVA with Kruskal-Wallis test.

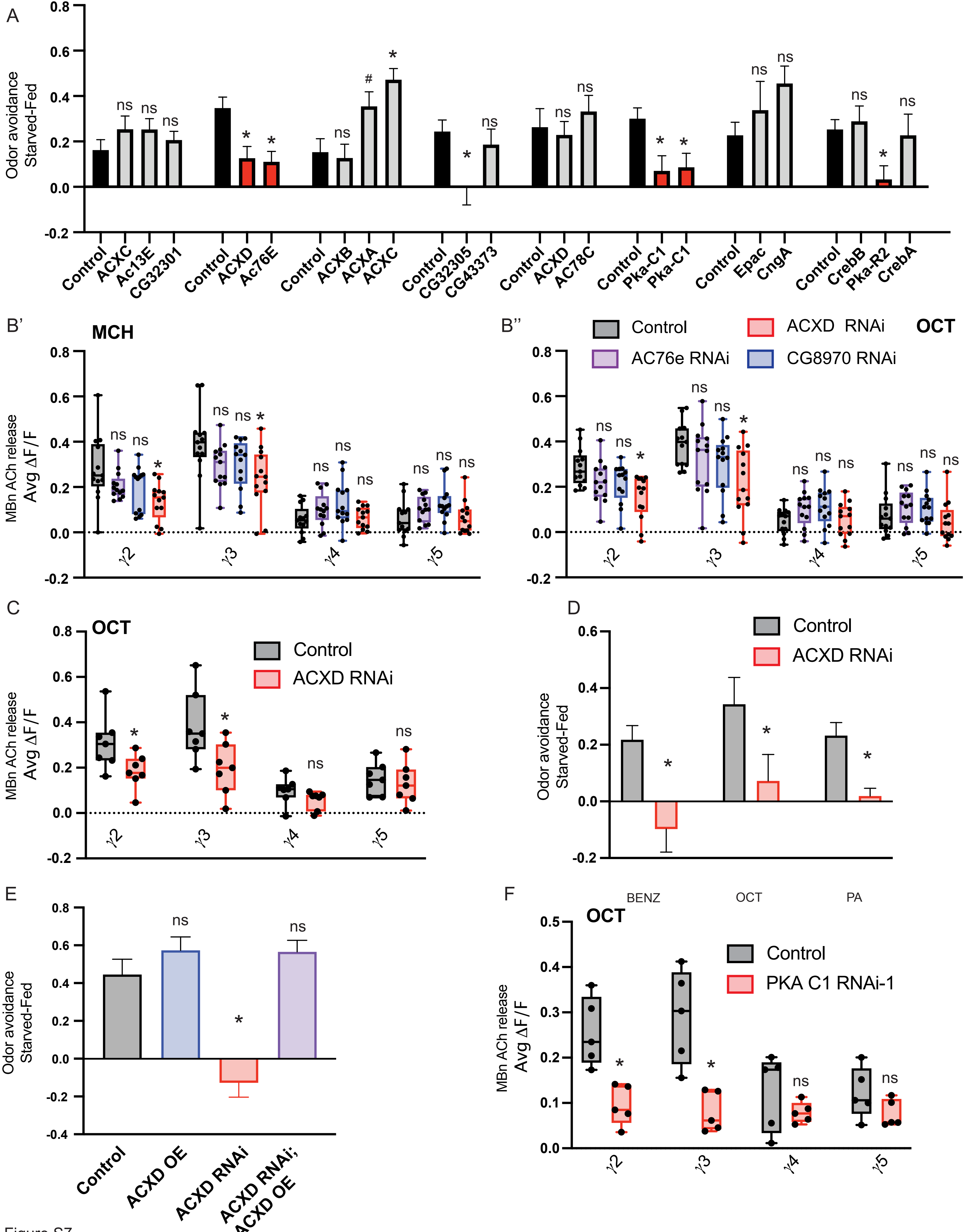

A
Odor avoidance Starved-Fed
Control
ACXC
Ac13E
CG32301
ACXD
Ac76E
ACXB
ACXA
CG32305
CG43373
AC78C
Pka-C1
Epac
CngA
CrebB
Pka-R2
CrebA
ns
*
#
B'
MCH
MBn ACh release Avg ΔF/F
B''
OCT
Control
ACXD RNAi
AC76e RNAi
CG8970 RNAi
γ2
γ3
γ4
γ5
C
D
E
ACXD OE
ACXD RNAi; ACXD OE
F
BENZ
PA
PKA C1 RNAi-1

Figure S7

Figure S7. cAMP signaling is required for state-dependent odor preferences and MB neurotransmission. Related to Figure 6. (A) Starved-Fed odor (MCH) avoidance scores for the re-screen. RNAi was expressed in MBn using R13F02-gal4. n=6-10 (B) The GRAB ACh sensor and RNAi were expressed in MBn using R13F02-gal4. Average odor responses were quantified for each compartment in γ2-5. n=6 (C) Replication experiment using only ACXD RNAi and control flies. The GRAB ACh sensor and RNAi were expressed in MBn using R13F02-gal4. Average odor responses were quantified for each compartment in γ2-5. n=7 (D) Starved-Fed odor avoidance scores. RNAi was expressed in MBn using R13F02-gal4. n=10 (E) Starved-Fed odor avoidance scores. RNAi was expressed in MBn using R13F02-gal4. n=8 (F) The GRAB ACh sensor and RNAi were expressed in MBn using R13F02-gal4. Average odor responses were quantified for each compartment in γ2-5. n=5-6 Box-and-whisker plots show the range of individual data points, with the interquartile spread as the box and the median as the line bisecting each box. *$P < 0.05$. (A-B, E) one-way ANOVA with Kruskal-Wallis test. (C-D, F) Mann-Whitney test.

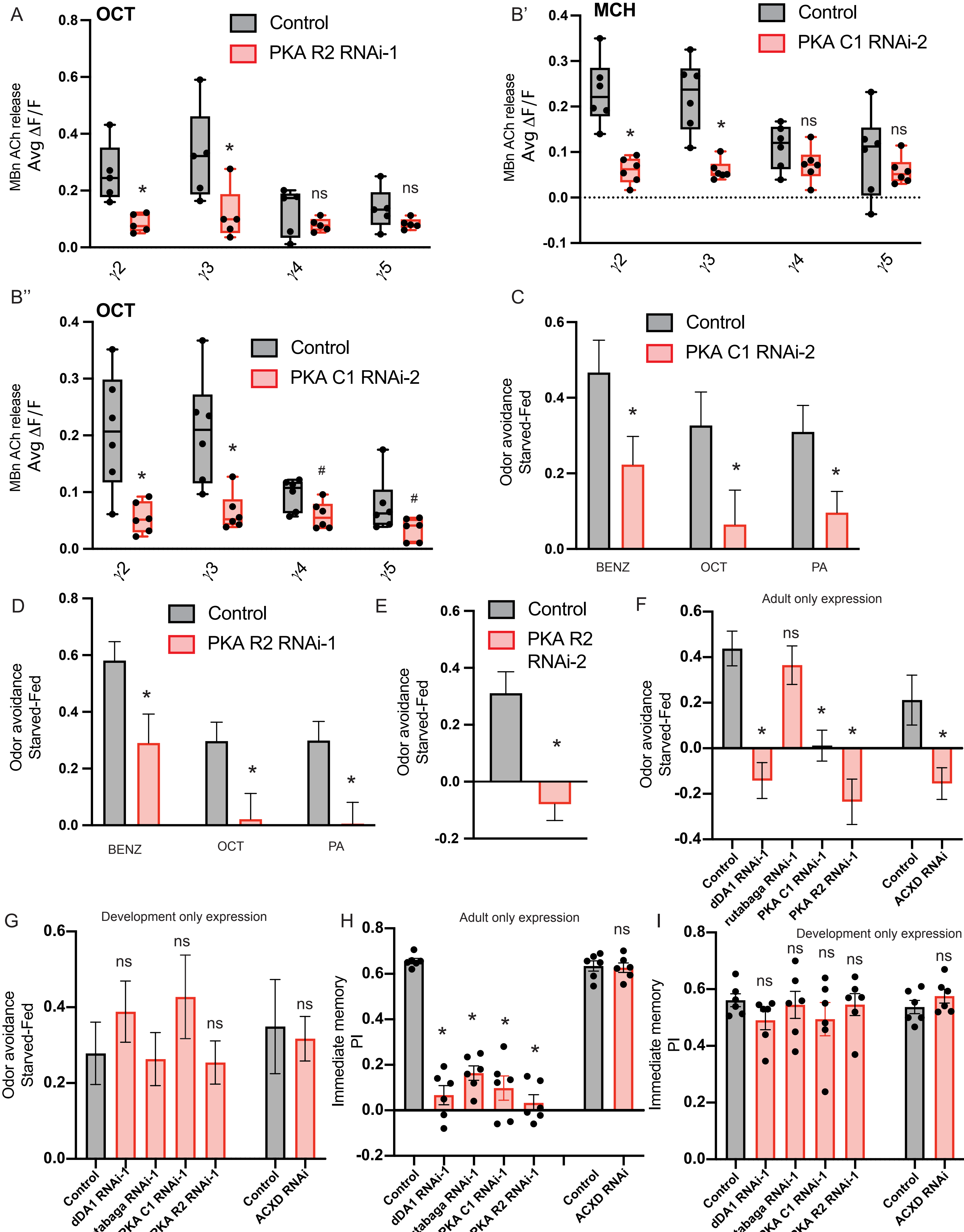
A
OCT
Control
PKA R2 RNAi-1
MBn ACh release
Avg ΔF/F
γ2
γ3
γ4
γ5
B'
MCH
PKA C1 RNAi-2
B''
OCT
C
Odor avoidance
Starved-Fed
BENZ
OCT
PA
D
E
PKA R2
RNAi-2
F
Adult only expression
dDA1 RNAi-1
rutabaga RNAi-1
PKA C1 RNAi-1
PKA R2 RNAi-1
ACXD RNAi
G
Development only expression
H
Immediate memory
PI
I

Figure S8

Figure S8. cAMP signaling is required for state-dependent odor preferences and MB neurotransmission. Related to Figure 6. (A-B) The GRAB ACh sensor and RNAi were expressed in MBn using R13F02-gal4. Average odor responses were quantified for each compartment in γ2-5. n=5-6 (C) Starved-Fed odor avoidance scores. RNAi was expressed in MBn using R13F02-gal4. n=10 (D) Starved-Fed odor avoidance scores. RNAi was expressed in MBn using R13F02-gal4. n=6-10 (E) Starved-Fed odor avoidance scores. RNAi was expressed in MBn using R13F02-gal4. n=8 (F-G) Starved-Fed odor (MCH) avoidance scores. RNAi was expressed in MBn using gal80ts;R13F02-gal4 n=6 (F) during adulthood only or (G) during development only. (H-I) Immediate memory PIs were calculated. RNAi was expressed in MBn using gal80ts;R13F02-gal4. n= 6 (H) during adulthood only or (I) during development only. Box-and-whisker plots show the range of individual data points, with the interquartile spread as the box and the median as the line bisecting each box. *$P < 0.05$. (A-E) Mann-Whitney test. (F-I) one-way ANOVA with Sidak's test.

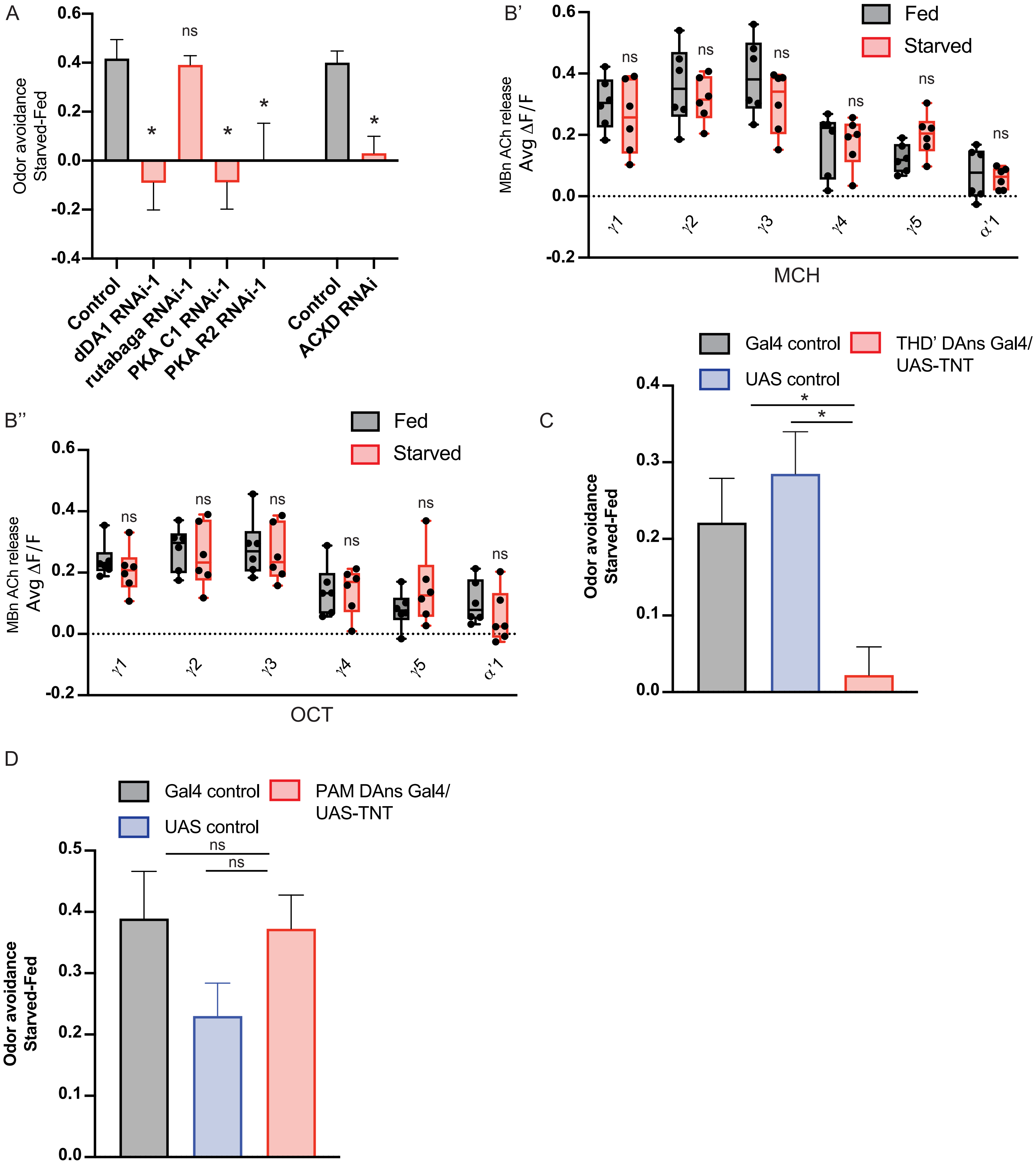
A
Odor avoidance Starved-Fed
ns
*
*
*
*
Control
dDA1 RNAi-1
rutabaga RNAi-1
PKA C1 RNAi-1
PKA R2 RNAi-1
Control
ACXD RNAi
B'
Fed
Starved
MBn ACh release Avg ΔF/F
ns
MCH
B''
OCT
C
Gal4 control
UAS control
THD' DAns Gal4/ UAS-TNT
D
PAM DAns Gal4/ UAS-TNT

Figure S9

Figure S9. Hunger engages the MB circuit in odor avoidance by facilitating MBON-γ2α'1 odor responses. Related to Figure 7. (A) Starved-Fed odor (MCH) avoidance scores. RNAi was expressed in γMBn using 1471-gal4. n=8 (B) The GRAB ACh sensor was expressed in MBn using R13F02-gal4. n=6 Average odor responses were quantified for each compartment in γ2-5 in fed and starved flies for (B') MCH and (B'') OCT. (C-D) Starved-Fed odor (MCH) avoidance scores. n=6 TNT was expressed in (C) PPL1 DANs using THD'-gal4 and (D) PAM DANS using R58E02-gal4. Box-and-whisker plots show the range of individual data points, with the interquartile spread as the box and the median as the line bisecting each box. $^*P < 0.05$. (A) one-way ANOVA with Dunnett's test. (B) Mann-Whitney test. (C,D) one-way ANOVA with Sidak's test.

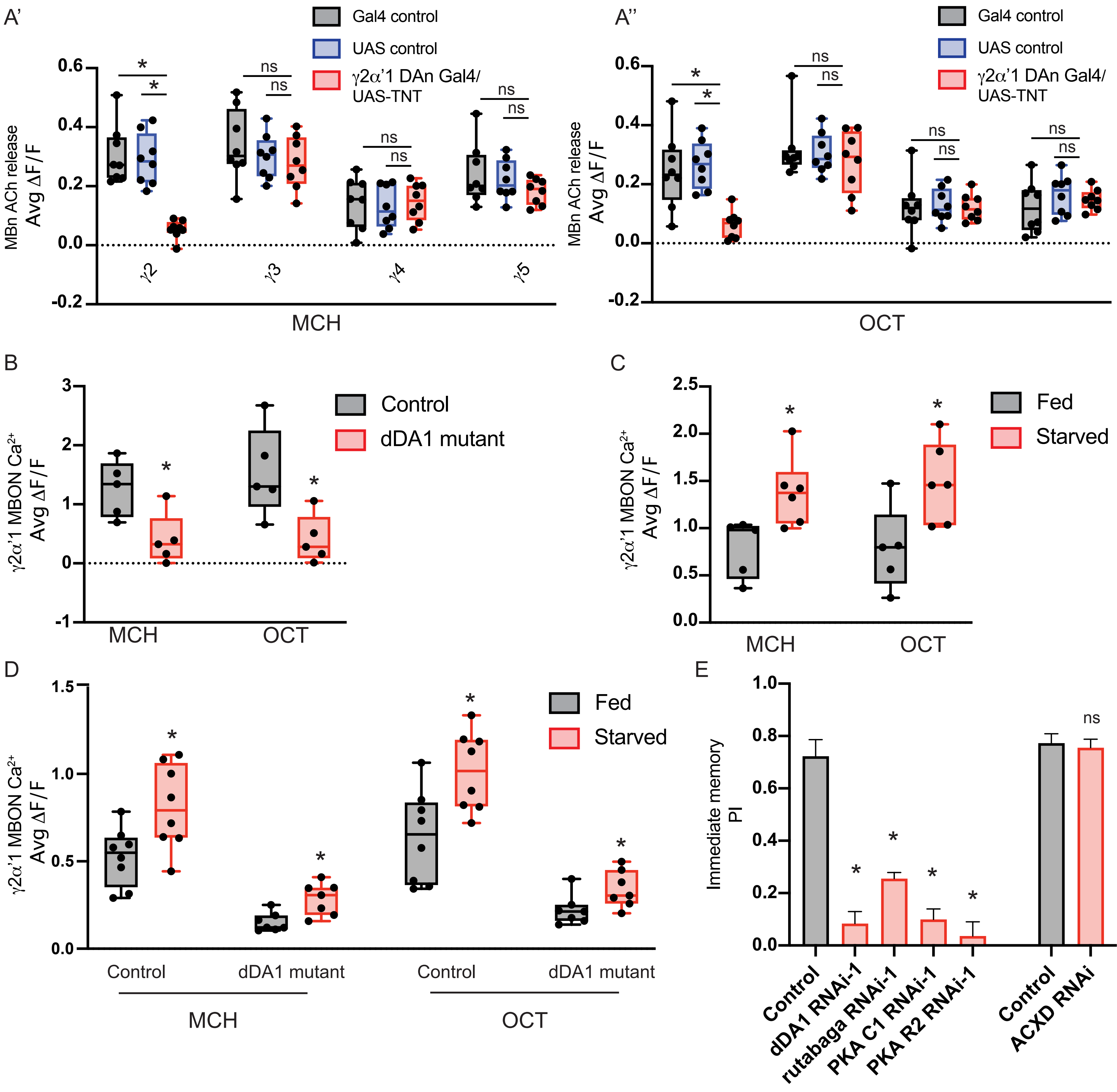
A'
Gal4 control
UAS control
γ2α'1 DAn Gal4/
UAS-TNT
MBn ACh release
Avg ΔF/F
γ2
γ3
γ4
γ5
MCH
A''
OCT
B
γ2α'1 MBON Ca2+
Avg ΔF/F
Control
dDA1 mutant
MCH
OCT
C
Fed
Starved
D
E
Immediate memory
PI
Control
dDA1 RNAi-1
rutabaga RNAi-1
PKA C1 RNAi-1
PKA R2 RNAi-1
ACXD RNAi
ns
*

Figure S10

Figure S10. Hunger engages the MB circuit in odor avoidance by facilitating MBON-γ2α'1 odor responses. Related to Figure 7.(A') MCH and (A'') OCT. GCaMP was expressed in MBON-γ2α'1 using R25D01-gal4. (B-C) GcaMP was expressed in MBON-γ2α'1 using R25D01-gal4. n=5-6. Average odor (MCH and OCT) responses were quantified for (B) dDA1 mutant and WT flies and (C) fed and starved flies. (D) GCaMP was expressed in MBON-γ2α'1 using R25D01-gal4. Average odor (MCH and OCT) responses were quantified. n=8 (E) Immediate memory PIs were calculated. RNAi was expressed in γMBn using 1471-gal4. n=6. Box-and-whisker plots show the range of individual data points, with the interquartile spread as the box and the median as the line bisecting each box. *P < 0.05. (A,B,C,D) Mann-Whitney test. (E) one-way ANOVA with Dunnett's test.

**Figure 5C**	**Control Fed**	**Control Starved**	**dda1 RNAi-1 Fed**	**dda1 RNAi-1 Starved**				
**MCH**	-0.46±0.1	-0.092±0.083	-0.58±0.048	-0.46±0.054				
**OCT**	-0.49±0.044	-0.14±0.077	-0.44±0.13	-0.42±0.063				
**BENZ**	-0.57±0.059	-0.21±0.11	-0.5±0.069	-0.42±0.066				
**PA**	-0.51±0.055	-0.25±0.055	-0.42±0.049	-0.37±0.051				
**Figure 5D**	**Control Fed**	**Control Starved**	**rut RNAi-1 Fed**	**rut RNAi-1 Starved**	**Control Fed**	**Control Starved**	**rut RNAi-2 Fed**	**rut RNAi-2 Starved**
	-0.14±0.063	0.18±0.076	-0.17±0.071	0.14±0.049	0.059±0.12	0.26±0.069	-0.079±0.08	0.26±0.088
**Supp. Figure 7B**	**Control Fed**	**Control Starved**	**dda1 RNAi-2 Fed**	**dda1 RNAi-2 Starved**	**Control Fed**	**Control Starved**	**dda1 mutant Fed**	**dda1 mutant Starved**
**MCH**	-0.2±0.049	0.12±0.086	-0.19±0.074	-0.21±0.095	-0.12±0.067	0.26±0.072	-0.14±0.051	-0.23±0.08
**Figure 6A**	**ACXB Fed**	**ACXB Starved**	**AC3 Fed**	**AC3 Starved**	**AC76E Fed**	**AC76E Starved**	**ACXC Fed**	**ACXC Starved**
**MCH**	-0.24±0.16	0.14±0.086	-0.14±0.1	0.11±0.069	-0.25±0.063	0.26±0.093	-0.16±0.076	0.083±0.048
	**AC76E Fed**	**AC76E Starved**	**CG43373 Fed**	**CG43373 Starved**	**CG32305 Fed**	**CG32305 Starved**	**ACXA Fed**	**ACXA Starved**
	-0.22±0.047	-0.023±0.056	-0.078±0.11	0.18±0.064	-0.39±0.099	0.2±0.12	-0.25±0.08	0.02±0.04
	**AC3 Fed**	**AC3 Starved**	**ACXD Fed**	**ACXD Starved**	**CG32301 Fed**	**CG32301 Starved**	**ACXC Fed**	**ACXC Starved**
	0.029±0.066	0.46±0.075	-0.063±0.079	0.14±0.049	-0.18±0.097	0.097±0.083	-0.062±0.085	0.089±0.076
	**ACXA Fed**	**ACXA Starved**	**CG32301 Fed**	**CG32301 Starved**	**CG32305 Fed**	**CG32305 Starved**	**ACXD Fed**	**ACXD Starved**
	-0.2±0.092	0.005±0.11	-0.26±0.12	0.082±0.094	-0.31±0.086	0.13±0.08	-0.15±0.061	0.23±0.055
	**CG43373 Fed**	**CG43373 Starved**	**ACXC Fed**	**ACXC Starved**	**CG32301 Fed**	**CG32301 Starved**	**AC78C Fed**	**AC78C Starved**
	-0.033±0.075	0.23±0.1	-0.075±0.1	-0.0025±0.076	-0.021±0.12	0.17±0.14	-0.21±0.11	0.08±0.057
	**AC78C Fed**	**AC78C Starved**	**AC13E Fed**	**AC13E Starved**	**CG32301 Fed**	**CG32301 Starved**	**CG32305 Fed**	**CG32305 Starved**
	-0.17±0.11	-0.0075±0.075	-0.11±0.11	-0.13±0.063	-0.15±0.18	0.015±0.13	-0.13±0.088	0.027±0.085
	**AC13E Fed**	**AC13E Starved**	**ACXA Fed**	**ACXA Starved**	**ACXB Fed**	**ACXB Starved**	**ACXD Fed**	**ACXD Starved**
	-0.21±0.069	-0.22±0.048	-0.062±0.13	0.32±0.089	-0.27±0.078	0.11±0.09	-0.28±0.082	-0.085±0.12
	**CG32301 Fed**	**CG32301 Starved**	**ACXB Fed**	**ACXB Starved**	**AC13E Fed**	**AC13E Starved**	**AC3 Fed**	**AC3 Starved**
	-0.13±0.12	0.098±0.13	-0.092±0.058	-0.095±0.066	-0.42±0.051	-0.018±0.11	-0.3±0.12	-0.0017±0.057
	**AKAP200 Fed**	**AKAP200 Starved**	**CNGL Fed**	**CNGL Starved**	**EPAC Fed**	**EPAC Starved**	**PKAC2 Fed**	**PKAC2 Starved**
	-0.11±0.09	0.22±0.065	-0.31±0.06	0.075±0.16	0.18±0.12	0.35±0.089	-0.13±0.14	0.33±0.088
	**PKAC2 Fed**	**PKAC2 Starved**	**CREBA FED**	**CREABA Starved**	**CNGA Fed**	**CNGA Starved**	**PKAR2 Fed**	**PKAR2 Starved**
	-0.13±0.14	0.33±0.088	-0.018±0.058	0.092±0.084	0.15±0.069	0.19±0.068	-0.078±0.086	0.27±0.069
	**CREBB**	**CREBB Starved**	**PKAC1 Fed**	**PKAC1 Starved**	**PKAC3 Fed**	**PKAC3 Starved**	**CREBA Fed**	**CREBA Starved**
	-0.12±0.06	0.3±0.064	-0.23±0.06	-0.047±0.089	-0.076±0.058	0.28±0.064	-0.26±0.079	0.13±0.079
	**PKAC2 Fed**	**PKAC2 Starved**	**PKAC1 Fed**	**PKAC1 Starved**	**PKAR1 Fed**	**PKAR1 Starved**	**CREBA Fed**	**CREBA Starved**
	-0.099±0.068	0.2±0.11	-0.005±0.086	0.03±0.095	-0.08±0.054	0.19±0.11	0.03±0.065	0.28±0.1
	**CNGB Fed**	**CNGB Starved**	**PKAR2 Fed**	**PKAR2 Starved**	**CNGB Fed**	**CNGB Starved**	**CREBB Fed**	**CREBB Starved**
	0.08±0.068	0.31±0.066	-0.058±0.12	-0.15±0.099	-0.39±0.07	0.19±0.061	0.17±0.14	0.16±0.067

**Supp. Figure 7A**	**Control Fed**	**Control Starved**	**ACXC Fed**	**ACXC Starved**	**AC13E Fed**	**AC13E Starved**	**CG32301 Fed**	**CG32301 Starved**
**MCH**	0.029±0.062	0.19±0.068	-0.056±0.063	0.2±0.098	-0.06±0.083	0.19±0.048	0.077±0.064	0.28±0.042
	**Control Fed**	**Control Starved**	**ACXD Fed**	**ACXD Starved**	**AC76E Fed**	**AC76E Starved**		
	-0.097±0.074	0.25±0.062	-0.042±0.066	0.084±0.082	-0.11±0.055	0.003±0.075		
	**Control Fed**	**Control Starved**	**ACXB Fed**	**ACXB Starved**	**ACXA Fed**	**ACXA Starved**	**ACXC Fed**	**ACXC Starved**
	0.076±0.083	0.23±0.084	-0.023±0.081	0.1±0.091	-0.042±0.11	0.31±0.07	-0.1±0.073	0.37±0.066
	**Control Fed**	**Control Starved**	**CG32305 Fed**	**CG32305 Starved**	**CG43373 Fed**	**CG43373 Starved**		
	-0.039±0.068	0.21±0.076	-0.013±0.077	-0.015±0.13	-0.13±0.11	0.058±0.077		
	**Control Fed**	**Control Starved**	**ACXD Fed**	**ACXD Starved**	**AC78C Fed**	**AC78C Starved**		
	-0.074±0.091	0.19±0.073	-0.085±0.05	0.14±0.067	-0.13±0.046	0.21±0.088		
	**Control Fed**	**Control Starved**	**PKAC1 Fed**	**PKAC1 Starved**	**PKAC1 Fed**	**PKAC1 Starved**		
	-0.035±0.041	0.27±0.052	-0.053±0.059	0.024±0.059	0.023±0.044	0.11±0.075		
	**Control Fed**	**Control Starved**	**EPAC Fed**	**EPAC Starved**	**CNGA Fed**	**CNGA Starved**		
	0.03±0.067	0.26±0.048	0.0083±0.076	0.35±0.16	0.085±0.087	0.54±0.065		
	**Control Fed**	**Control Starved**	**CREBB Fed**	**CREBB Starved**	**PKAR2 Fed**	**PKAR2 Starved**	**CREBA Fed**	**CREBA Starved**
	-0.1±0.048	0.15±0.038	-0.16±0.068	0.13±0.067	-0.12±0.057	-0.083±0.063	-0.098±0.11	0.13±0.074
**Supp. Figure 7D**	**Control Fed**	**Control Starved**	**ACXD RNAi Fed**	**ACXD RNAi Starved**				
**BENZ**	-0.5±0.053	-0.28±0.045	-0.42±0.091	-0.52±0.071				
**OCT**	**-0.39±0.098**	**-0.049±0.091**	**-0.44±0.11**	**-0.37±0.071**				
**PA**	-0.39±0.035	-0.15±0.055	-0.34±0.019	-0.32±0.033				
**Supp. Figure 7E**	**Control Fed**	**Control Staved**	**ACXD OE Fed**	**ACXD OE Starved**	**ACXD RNAi Fed**	**ACXD Starved**	**ACXD RNAi;OE Fed**	**ACXD RNAi;OE Starved**
**MCH**	-0.53±0.066	-0.087±0.094	-0.45±0.061	0.12±0.08	-0.52±0.09	-0.65±0.057	-0.65±0.054	-0.082±0.068
**Supp. Figure 8C**	**Control Fed**	**Control Starved**	**PKAC1 RNAi-2 Fed**	**PKAC1 RNAi-2 Starved**				
**BENZ**	-0.47±0.064	-0.0051±0.1	-0.42±0.071	-0.2±0.078				
**OCT**	-0.41±0.1	-0.084±0.074	-0.45±0.085	-0.39±0.096				
**PA**	-0.24±0.067	0.073±0.073	-0.18±0.05	-0.081±0.061				
**Supp. Figure 8D**	**Control Fed**	**Control Starved**	**PKAR2 RNAi-1 Fed**	**PKAR2 RNAi-1 Starved**				
**BENZ**	-0.61±0.064	-0.027±0.069	-0.58±0.11	-0.28±0.091				
**OCT**	-0.34±0.072	-0.047±0.061	-0.39±0.095	-0.37±0.085				
**PA**	-0.35±0.06	-0.054±0.074	-0.32±0.092	-0.31±0.053				
**Supp. Figure 8F**	**Control Fed**	**Control Starved**	**dda1 RNAi-1 Fed**	**dda1 RNAi-1 Starved**	**rutabaga RNAi-1 Fed**	**rutabaga RNAi-1 Starved**	**PKA C1 RNAi-1 Fed**	**PKA C1 RNAi-1 Starved**
**MCH**	-0.44±0.049	0.0033±0.097	-0.31±0.047	-0.45±0.1	-0.43±0.047	-0.067±0.11	-0.37±0.054	-0.36±0.079
	**PKA R2 RNAi-1 Fed**	**PKA R2 RNAi-1 Starved**	**Control Fed**	**Control Starved**	**ACXD RNAi Fed**	**ACXD RNAi Starved**		
	-0.4±0.092	-0.64±0.11	-0.32±0.04	-0.11±0.15	-0.39±0.066	-0.54±0.073		
**Supp. Figur 8G**	**Control Fed**	**Control Starved**	**dda1 RNAi-1 Fed**	**dda1 RNAi-1 Starved**	**rutabaga RNAi-1 Fed**	**rutabaga RNAi-1 Starved**	**PKA C1 RNAi-1 Fed**	**PKA C1 RNAi-1 Starved**
**MCH**	-0.25±0.053	0.027±0.1	-0.27±0.085	0.11±0.076	-0.18±0.055	0.081±0.083	-0.23±0.11	0.2±0.11
	**PKA R2 RNAi-1 Fed**	**PKA R2 RNAi-1 Starved**	**Control Fed**	**Control Starved**	**ACXD RNAi Fed**	**ACXD RNAi Starved**		
	-0.14±0.074	0.12±0.033	-0.2±0.084	0.15±0.15	-0.23±0.077	0.089±0.032		

**Figure 7A**	**GAL4 Control Fed**	**GAL4 Control Starved**	**UAS Control Fed**	**UAS Control Starved**	**γ2α'21 DAn /TNT Fed**	**γ2α'21 DAn /TNT Starved**		
**MCH**	-0.38±0.063	-0.12±0.047	0.037±0.073	0.34±0.09	-0.085±0.085	-0.17±0.07		
**Figure 7E**	**GAL4 Control Fed**	**GAL4 Control Starved**	**UAS Control Fed**	**UAS Control Starved**	**γ2α'21 MBON /TNT Fed**	**γ2α'21 MBON/TNT Starved**		
**MCH**	-0.16±0.07	0.3±0.062	-0.29±0.079	0.16±0.038	-0.18±0.043	-0.057±0.09		
**Figure 7F**	**Control Fed**	**Control Starved**	**nACHRα1 RNAi Fed**	**nACHRα1 RNAi Starved**	**nACHRα2 RNAi Fed**	**nACHRα2 RNAi Starved**	**nACHRα3 RNAi Fed**	**nACHRα3 RNAi Starved**
**MCH**	-0.3±0.059	0.055±0.059	-0.17±0.033	0.065±0.065	-0.17±0.05	-0.18±0.027	-0.31±0.051	0.077±0.07
	**nACHRα4 RNAi Fed**	**nACHRα4 RNAi Starved**	**nACHRα5 RNAi Fed**	**nACHRα5 RNAi Starved**	**nACHRα6 RNAi Fed**	**nACHRα6 RNAi Starved**	**nACHRα7 RNAi Fed**	**nACHRα7 RNAi Starved**
	-0.15±0.078	0.11±0.061	-0.18±0.08	0.091±0.07	-0.23±0.042	0.047±0.072	-0.16±0.061	0.029±0.048
**Supp. Fig 9A**	**Control Fed**	**Control Starved**	**dda1 RNAi-1 Fed**	**dda1 RNAi-1 Starved**	**rutabaga RNAi-1 Fed**	**rutabaga RNAi-1 Starved**	**PKAC1 RNAi-1 Fed**	**PKAC1 RNAi-1 Starved**
**MCH**	-0.35±0.095	0.069±0.053	-0.24±0.037	-0.33±0.15	-0.24±0.039	0.15±0.035	-0.28±0.037	-0.37±0.15
	**PKAR2 RNAi-1 Fed**	**PKAR2 RNAi-1 Starved**	**Control Fed**	**Control Starved**	**ACXD RNAi Fed**	**ACXD RNAi Starved**		
**MCH**	-0.26±0.023	-0.26±0.21	-0.26±0.056	0.14±0.039	-0.33±0.026	-0.3±0.094		
**Supp. Fig 9C**	**GAL4 Control Fed**	**GAL4 Control Starved**	**UAS Control Fed**	**UAS Control Starved**	**THD' DAn /TNT Fed**	**THD' DAn /TNT Starved**		
**MCH**	-0.2±0.07	0.025±0.042	-0.012±0.063	0.27±0.045	-0.073±0.037	-0.051±0.037		
**Supp. Fig 9D**	**GAL4 Control Fed**	**GAL4 Control Starved**	**UAS Control Fed**	**UAS Control Starved**	**PAM DAn/TNT Fed**	**PAM DAn/TNT Starved**		
**MCH**	-0.039±0.067	0.35±0.085	0.14±0.045	0.37±0.061	0.15±0.056	0.52±0.055		

Table S1. Fed and starved odor avoidance scores. Related to Figures 5,6, and 7.
Mean PI scores shown with S.E.M.
